# Microclimate Comfort of Elevated Bridge Underpass Spaces Based on Sound and Thermal Environments: A Case Study of Fuzhou and Xiamen, Fujian Province

**DOI:** 10.3390/plants15192932

**Published:** 2026-09-24

**Authors:** Xinyu Zhang, Shuhong Huang, Yanbing Chen, Yiwei Han, Wenhao Liu, Wei Ren, Donghui Peng

**Affiliations:** College of Landscape Architecture and Art, Fujian Agriculture and Forestry University, Fuzhou 350100, China; 52419052045@fafu.edu.cn (X.Z.); huangshuhong@fafu.edu.cn (S.H.); 12319075021@fafu.edu.cn (Y.C.); 62519052003@fafu.edu.cn (Y.H.); 52419052096@fafu.edu.cn (W.L.)

**Keywords:** viaduct underpass space, microclimate regulation, thermal comfort, acoustic comfort, overall comfort, plant landscape, urban morphology, environmental simulation, hot–humid climate

## Abstract

In the context of urban renewal, spaces beneath elevated bridges are gradually becoming important public activity areas, with their microclimate and landscape quality influenced by multiple factors including bridge structure, vegetation configuration, and traffic conditions. This study focuses on 12 typical elevated bridge underpass spaces in Fuzhou and Xiamen, two hot–humid subtropical cities in China, and integrates field environmental monitoring, questionnaire surveys, spatial landscape metrics, and statistical analysis to explore how plant landscape factors are significantly associated with thermal and acoustic conditions and overall comfort levels. Results show the following: (1) Overall comfort refers to the overall subjective evaluation of the elevated bridge underpass environment under combined thermal and acoustic conditions. Thermal and acoustic comfort jointly explained 71.3% of its variance (*R*^2^ = 0.713), with thermal comfort contributing more strongly. (2) Bridge orientation was associated with differences in shading conditions and microclimatic characteristics, with east–west-oriented elevated bridge underpass spaces generally offering better overall comfort. (3) Green coverage rate, green buffer width, distance from roads, and hard paving proportion were identified as key landscape factors associated with overall comfort. Controlled regression showed significant associations, with green coverage rate showing the strongest positive association with OCV (*β* = 0.612), whereas hard paving proportion showed the strongest negative association (*β* = −0.628); the models explained 66.8–68.4% of OCV variance. (4) Appropriate plant landscape configurations were associated with more favorable microclimatic conditions under bridges. Within the studied sites, higher comfort was associated with approximately 40–55% green coverage, 8–15 m green-buffer width, a road distance above 60 m, and a hard paving ratio below 50%. These values should be regarded as context-specific design references derived from the observed regression patterns rather than universal optimal thresholds. This study identifies associations among plant landscape configuration, microclimate, and environmental comfort in elevated bridge underpass spaces based on field evidence from Fuzhou and Xiamen, providing context-specific references for landscape renewal in similar hot–humid subtropical settings.

## 1. Introduction

Elevated transportation infrastructure has generated extensive spaces beneath and alongside viaducts in urban areas [1]. Due to their unique spatial conditions and functional potential, elevated bridge underpass spaces have increasingly attracted attention as potential sites for public space regeneration and improvement [2]. These spaces have been explored for public space regeneration, pedestrian connectivity, and ecological improvement [3,4,5]. Recent studies have further examined their regeneration through landscape design, greening, public activities, and environmental improvement [5,6,7,8]. However, compared with functional and spatial renewal, the environmental performance and comfort mechanisms of elevated bridge underpass spaces remain insufficiently understood.

Unlike commonly studied semi-open environments, elevated bridge underpass spaces are characterized by the simultaneous effects of large overhead structures, constrained airflow, and continuous exposure to adjacent traffic. The bridge structure reduces direct solar radiation and creates relatively stable shaded zones, while restricted air exchange, high humidity, and localized heat accumulation may still cause thermal discomfort. At the same time, their frequent proximity to major urban roads and expressways exposes users to persistent traffic noise. The World Health Organization (WHO) indicates that continuous noise exposure not only causes annoyance and sleep disturbance but also increases health risks such as ischemic heart disease and hypertension [9]. According to the 2024 China Noise Pollution Prevention and Control Report, although urban acoustic environment management continues to progress, traffic noise remains a significant source of pressure in sound environment control [10]. Thermal stress and noise exposure jointly affect users’ overall environmental perception and may constrain the public service functions of elevated bridge underpass spaces. Therefore, understanding the coupled thermal and acoustic conditions of these spaces is important for improving their environmental quality.

Outdoor thermal comfort studies have mainly applied integrated indices such as the Physiological Equivalent Temperature (PET) and Universal Thermal Climate Index (UTCI) to evaluate the relationships between environmental conditions and human thermal perception. Recent studies have demonstrated that thermal comfort thresholds vary substantially across climatic contexts [11,12,13,14,15]. For example, a field study in Xiamen, a humid subtropical city in southeastern China, reported neutral PET values of 19.3 °C in spring and 24.7 °C in summer, with a 90% thermal acceptability limit below 32.8 °C in the summer [16,17,18,19]. Existing studies have shown that urban morphology, greenery configuration, shading conditions, and ventilation environments significantly influence human thermal comfort levels [16,17,18,19]. A systematic review of 76 peer-reviewed studies found a generally positive association between urban green infrastructure and pedestrian thermal comfort across different climate types [20]. However, the magnitude of this effect varied with climatic and spatial conditions. Outdoor thermal comfort is influenced not only by physical environmental conditions but also by behavioral adaptation and psychological factors [21]. Consequently, integrating objective environmental monitoring with subjective questionnaire surveys has become a key methodological framework in thermal comfort research [21,22,23,24]. Traffic is one of the dominant sources of urban outdoor noise, and the acoustic conditions of activity spaces are strongly influenced by the spatial relationship between activity areas and surrounding roads. Sound levels generally decrease with increasing distance from traffic sources. However, road configuration and surrounding built form can modify traffic noise propagation [25,26]. Elevated road infrastructure can further modify traffic noise distribution. A field measurement study in Guangzhou found that elevated bridge underpass spaces experienced higher noise exposure than areas above the viaduct, while road distance and noise barriers affected spatial noise patterns [27]. Therefore, acoustic comfort assessment increasingly combines objective indicators such as the A-weighted equivalent continuous sound pressure level (L_Aeq_) with subjective evaluations of acoustic sensation and comfort [28,29,30]. Moreover, sound environment quality depends not only on sound pressure levels but also on factors such as sound source types, spatial context, and usage purposes [31,32]. Therefore, objective acoustic indicators such as L_Aeq_ should be considered together with subjective acoustic evaluations when assessing outdoor acoustic comfort [33,34,35,36,37]. Evidence from outdoor park environments further indicates that thermal perception can alter acoustic perception. Mohammadzadeh et al. found that higher TSV and PET were associated with lower soundscape quality and overall impression, whereas higher TCV was positively associated with these acoustic evaluations [38].

Recent studies have increasingly demonstrated that thermal and acoustic environments interact in shaping outdoor environmental perception, rather than affecting comfort independently [39]. Field evidence has further confirmed this interaction. Lin et al. [40] found that excessive acoustic exposure could worsen thermal perception. Conversely, increasing PET altered acoustic perception. Notably, the most comfortable acoustic perception was reported at a PET of approximately 30.1 °C, indicating a bidirectional interaction between thermal and acoustic perceptions [40]. Recent studies have increasingly shifted from single-environment assessments toward integrated evaluations of thermal–acoustic interactions and overall comfort. Field studies have shown that thermal and acoustic conditions jointly influence subjective comfort, while their relative contributions vary with environmental conditions and spatial contexts [16,41]. For example, Jin et al. found that thermal and acoustic environments jointly explained 58.7–68.2% of the variation in overall comfort in urban squares. Reference [42] further showed that overall comfort remained consistently low when L_Aeq_ exceeded 56 dBA, regardless of UTCI [43]. These findings indicate that integrating objective environmental indicators with subjective comfort evaluations provides a more comprehensive framework for assessing outdoor environmental quality.

Despite advances in thermal and acoustic comfort research, the environmental comfort mechanisms of elevated bridge underpass spaces remain insufficiently understood. Existing studies have mainly focused on spatial regeneration, landscape improvement, or individual environmental factors, while the coupled effects of thermal and acoustic environments in elevated bridge underpass spaces have received limited attention. Due to the combined influence of overhead structures, constrained airflow, and traffic-related disturbances, these spaces require a targeted evaluation framework rather than directly applying conventional outdoor comfort models.

Therefore, this study investigates the microclimate comfort of elevated bridge underpass spaces based on thermal and acoustic environments. Twelve representative elevated bridge underpass spaces in Fuzhou and Xiamen, China, were selected as case studies. Field measurements, subjective questionnaires, and environmental simulations were combined to explore the relationships among thermal conditions, acoustic environments, and overall comfort perception. The objectives of this study are to: (1) evaluate the thermal and acoustic comfort characteristics of elevated bridge underpass spaces; (2) identify the influencing pathways of thermal and acoustic factors on overall comfort; and (3) reveal the potential effects of spatial and landscape characteristics on environmental comfort improvement.

## 2. Research Results

### 2.1. Analysis of the Objective and Subjective Mechanisms of the Thermal–Acoustic Environment

#### 2.1.1. Correlation Analysis Between Thermal–Acoustic Environment Parameters and Subjective Evaluation

Using IBM SPSS Statistics (Version 27.0) software for Spearman rank correlation analysis, the correlation between thermal–acoustic environment parameters and subjective perception evaluation was tested. (Table 1) Air temperature, mean radiant temperature, and PET were significantly correlated with thermal perception indicators (*p* < 0.01, |*R*| = 0.27–0.62). Wind speed and relative humidity showed weaker correlations (|*R*| = 0.10–0.38) and were not significantly related to thermal expectations. The stronger correlation between PET and mean radiant temperature suggests that composite thermal indices may better represent subjective thermal perception. The acoustic environment parameter L_Aeq_ was significantly negatively correlated with thermal comfort (*p* < 0.05, |*R*| = 0.17–0.48) but had no significant relationship with thermal expectations.

For acoustic environment perception, L_Aeq_ was significantly negatively correlated with ASV, ACV, and APV (*p* < 0.01, |*R*| = 0.45–0.60). Some thermal environment parameters also had certain correlations with acoustic perception but overall, they were weaker than those of L_Aeq_. Air temperature had a lower correlation with acoustic perception, and wind speed and relative humidity had no significant correlation. Average radiant temperature and PET had moderate correlations with acoustic comfort, subjective loudness, and acoustic expectations (*p* < 0.05). These results indicate that L_Aeq_ is the most representative indicator of acoustic perception, while the other environmental parameters were retained to examine potential thermal–acoustic interactions rather than as core acoustic indicators.

OCV analysis showed that air temperature, average radiant temperature, and PET were moderately negatively correlated with overall perception (*p* < 0.01, |*R*| = 0.31–0.44), while wind speed and relative humidity reached significance but had a weak correlation (|*R*| < 0.15); L_Aeq_ was significantly negatively correlated with overall perception. Overall, PET and L_Aeq_ showed relatively stronger relationships with subjective evaluations and were therefore selected as the core indicators for subsequent thermal and acoustic analyses, respectively.

#### 2.1.2. Correlation Analysis Between Thermal–Acoustic Comfort and Overall Comfort

To further examine the relationships among thermal comfort, acoustic comfort, and overall comfort, Spearman rank correlation analysis was conducted on TCV, ACV, and OCV (Table 2). The results showed significant associations of TCV and ACV with OCV, providing statistical support for their inclusion in the overall comfort model. The analysis showed that overall comfort is not associated with a single environmental factor, but that it is a combined result of thermal and acoustic environments. These correlations help clarify how thermal and acoustic comfort contribute to overall environmental perception. They also provide a basis for constructing the overall comfort evaluation model.

To examine whether respondent characteristics affected the main comfort relationships, a supplementary subgroup analysis was conducted using gender, age, and outdoor stay duration. Spearman correlations between TCV/ACV and OCV were calculated for each subgroup (Table 3). The associations remained generally consistent in direction and significance across the subgroups, indicating that the main relationships were robust to demographic and behavioral differences.

### 2.2. Characteristics of the Thermal–Acoustic Environment and Overall Comfort in Spaces Under Elevated Bridges

**Figure 1:**
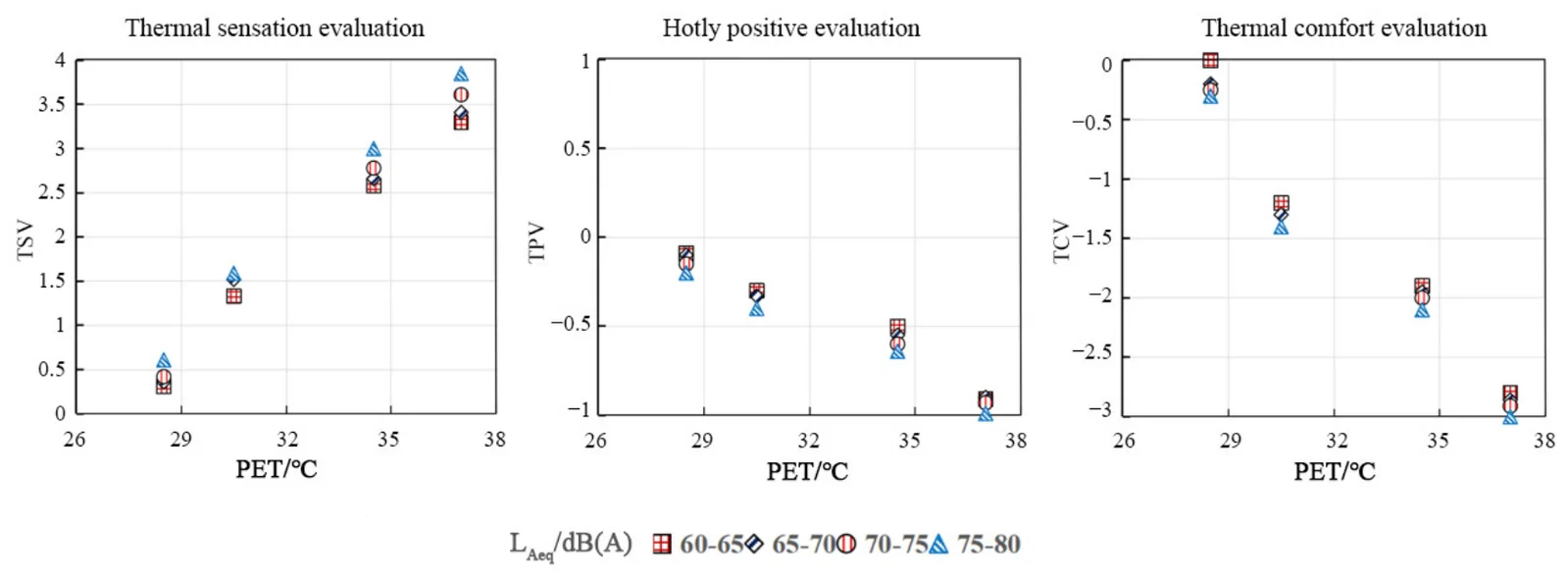
Thermal environment perception evaluation of respondents.

The thermal environment monitoring results showed that although the bridge structure can effectively weaken solar shortwave radiation, the elevated bridge underpass space is still generally in a high thermal stress state. PET was significantly positively correlated with respondents’ TSV, while it was significantly negatively correlated with thermal comfort vote (TCV). As PET increased, TSV increased and TCV decreased significantly (Figure 1), indicating that higher thermal exposure directly corresponded to stronger heat perception and lower thermal comfort. This suggests that bridge shading alone was insufficient to offset thermal stress under hot–humid summer conditions.

Further analysis reveals that PET was associated not only with thermal sensation and thermal comfort evaluations but also influences respondents’ perception of other environmental factors. When PET reaches a high level, respondents’ evaluations of the environmental quality tend to be negative, showing higher sensitivity and lower environmental tolerance.

**Figure 2:**
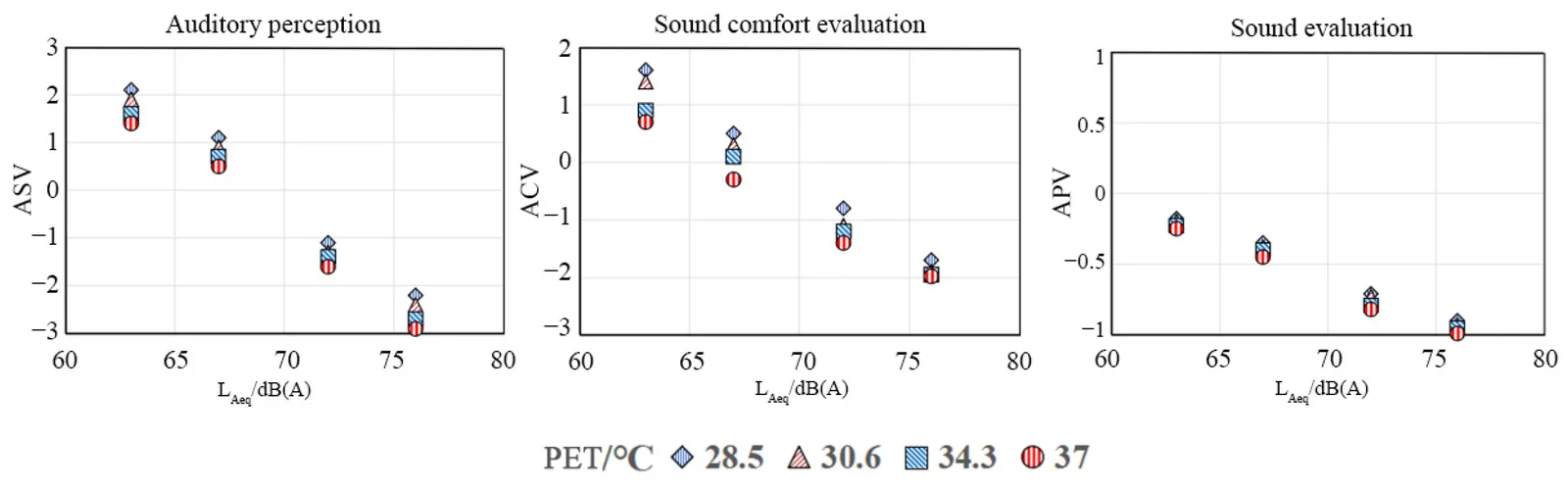
Evaluation of respondents’ perception of the acoustic environment.

The sound environment monitoring results show that the sound environment of the elevated bridge underpass space is mainly affected by the adjacent traffic roads. Most sample points have L_Aeq_ values in the medium to high range, and traffic noise becomes an important factor affecting the spatial experience. As L_Aeq_ increases, respondents’ ASV continues to rise, while ACV continues to decline (Figure 2). Particularly at sample points with high traffic flow, continuous vehicle operation noise has become an important environmental constraint limiting the quality of the space.

**Figure 3:**
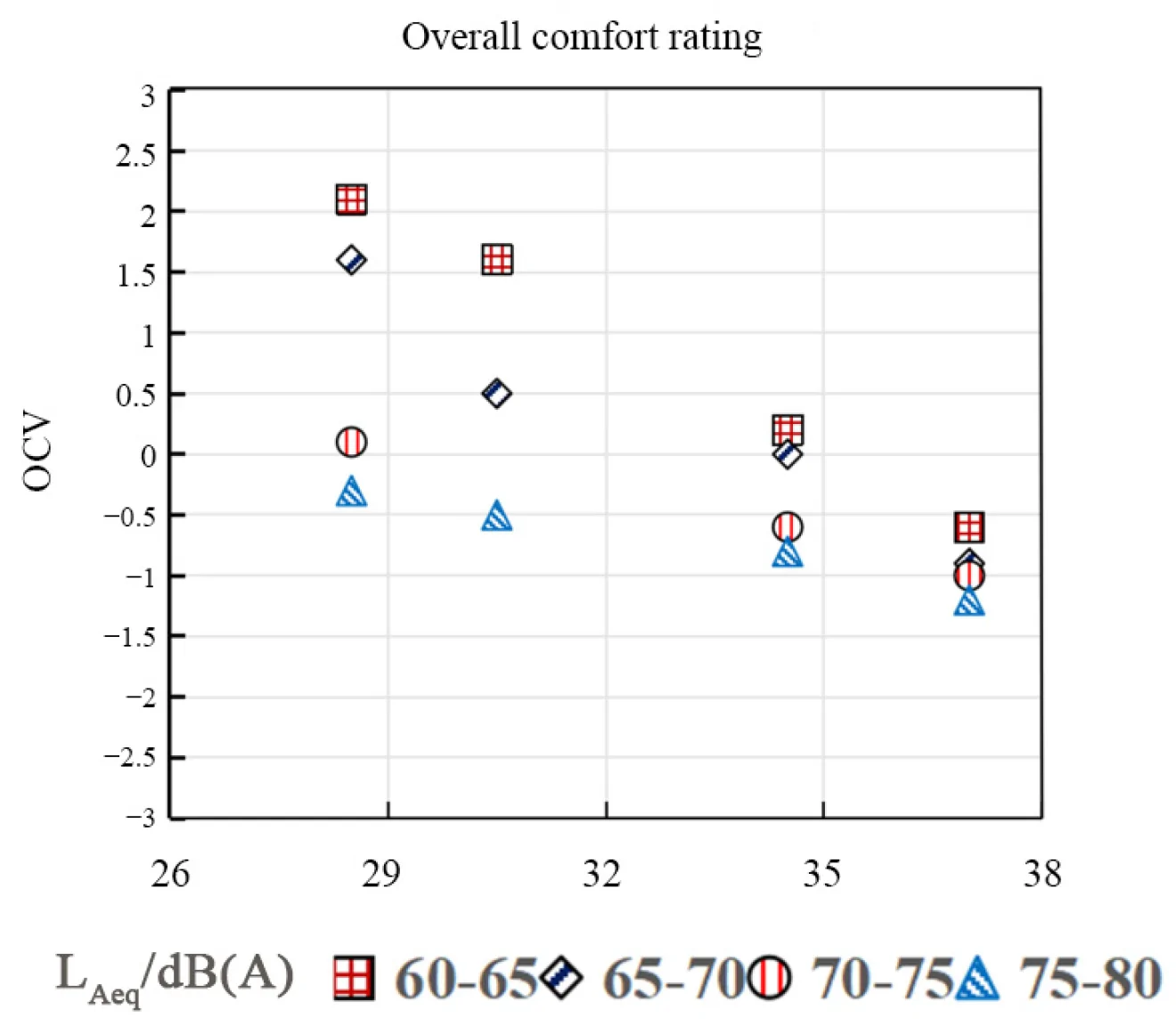
Overall comfort rating of respondents.

Respondents showed different acoustic comfort evaluations under different thermal conditions at the same noise level. When PET was high, respondents were more likely to report negative evaluations of noise. At lower thermal stress levels, the same L_Aeq_ corresponded to higher acoustic comfort. This pattern suggests an interaction between thermal and acoustic conditions, which was further examined using the two-way ANOVA. The overall comfort analysis results further verify this feature. As PET and L_Aeq_ increase simultaneously, the overall comfort evaluation continues to decline. When PET is low and L_Aeq_ is controlled at a lower level, respondents generally give higher overall comfort evaluations; while under the combined effect of high temperature and high noise, the overall comfort level drops rapidly (Figure 3).

**Figure 4:**
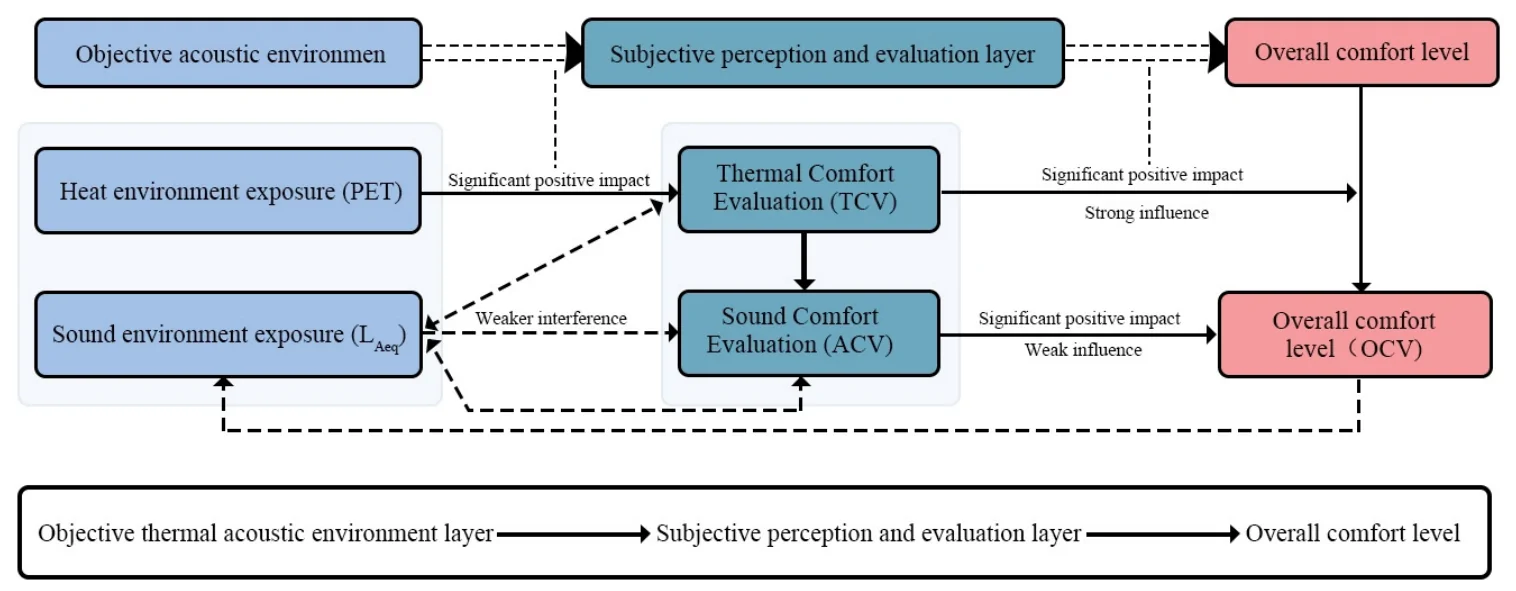
Conceptual framework of thermal–acoustic comfort relationships in elevated bridge underpass spaces.

Thermal and acoustic comfort jointly explained 71.3% of the variance in OCV (*R*^2^ = 0.713), with thermal comfort showing the stronger contribution. The observed correlations were consistent with two conceptual pathways, namely “PET–TSV–TCV” and “L_Aeq_–ASV–ACV”, which were used to summarize the thermal and acoustic comfort relationships (Figure 4). These pathways represent an interpretive framework based on the correlation and regression results rather than statistically tested mediation effects.

### 2.3. The Impact of Bridge Structure Form on Overall Comfort

The bridge structure form is an important factor determining the basic conditions of the elevated bridge underpass space. This study focuses on analyzing the impact of the bridge orientation, width-to-height ratio, and adjacent road conditions on overall comfort. The results indicate that bridge orientation is the main structural factor associated with differences in environmental quality and overall comfort. The 12 sample points can be classified into three types: east–west orientation, north–south orientation, and northeast–southwest orientation, as shown in Table 4.

The 12 sites showed clear differences in overall comfort across orientations (Table 5). OCV ranged from −0.58 to 0.12, with east–west-oriented spaces generally showing lower PET and higher OCV than north–south and northeast–southwest-oriented spaces. Lower OCV was generally associated with higher thermal or acoustic exposure, with thermal differences being more pronounced.

The influence of the aspect ratio on the OCV has strong context dependence, and its effect is influenced by the configuration of different scale plant landscapes and the surrounding spatial environment. The research results show that the plant landscape regulates the microclimate processes such as shading, ventilation, and sound environment, and has an important impact on the comfort level of the bridge underpass space. At the same time, traffic noise is an important external factor that restricts the environmental quality, and the multi-level vegetation configuration helps to weaken noise propagation and improve overall comfort. However, these differences may also be partly related to site-specific characteristics, such as vegetation, road exposure, and spatial enclosure.

### 2.4. The Influence of Microscopic Landscape Elements on the Overall Comfort Level

To identify the key landscape factors associated with OCV, Spearman correlation and controlled regression analyses were conducted using the measurement point data.

#### 2.4.1. The Correlation Between Microscopic Landscape Elements and Overall Comfort Level

The results of the correlation analysis show that the microscopic landscape elements affecting the overall comfort level under different bridge orientation conditions have certain commonalities, but also significant differences. To further assess potential redundancy among the candidate predictors, Spearman correlations among the landscape variables were examined before regression modeling (Table 6).

**Figure 5:**
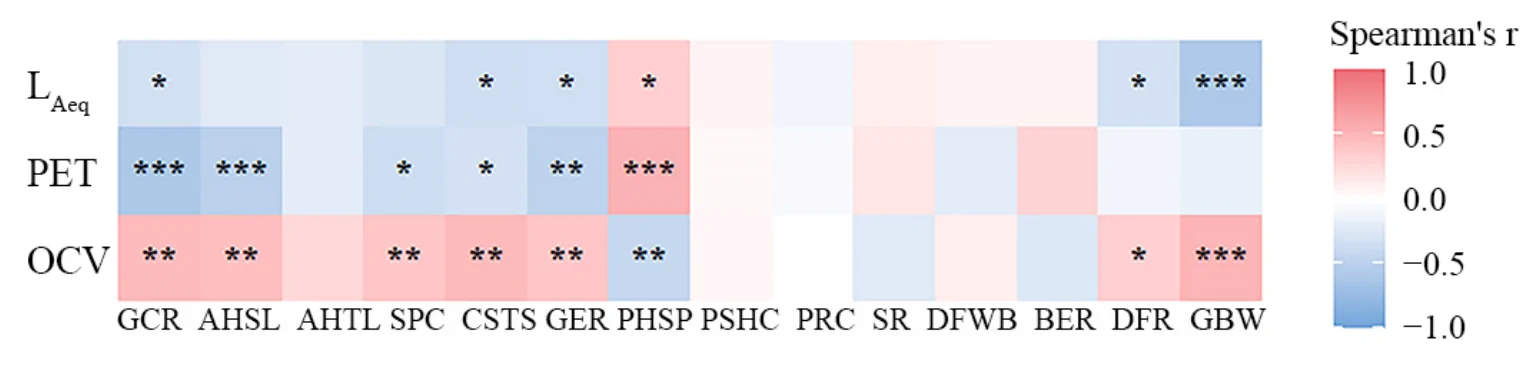
Correlation coefficients of key micro-landscape factors in the north–south/east–northwest to south–southwest orientation group. Note: * *p* < 0.05, ** *p* < 0.01, and *** *p* < 0.001.

In the north–south/northeast–southwest group, green buffer width, tree canopy size, green coverage rate, and shrub-layer height were positively correlated with OCV (*p* < 0.01). In contrast, hard paving proportion was negatively correlated with OCV (ρ = −0.423, *p* < 0.01). Green coverage rate and average height of the shrub layer are significantly negatively correlated with PET, while the green buffer width and tree canopy size are significantly negatively correlated with L_Aeq_. (Table 7, Figure 5)

**Figure 6:**
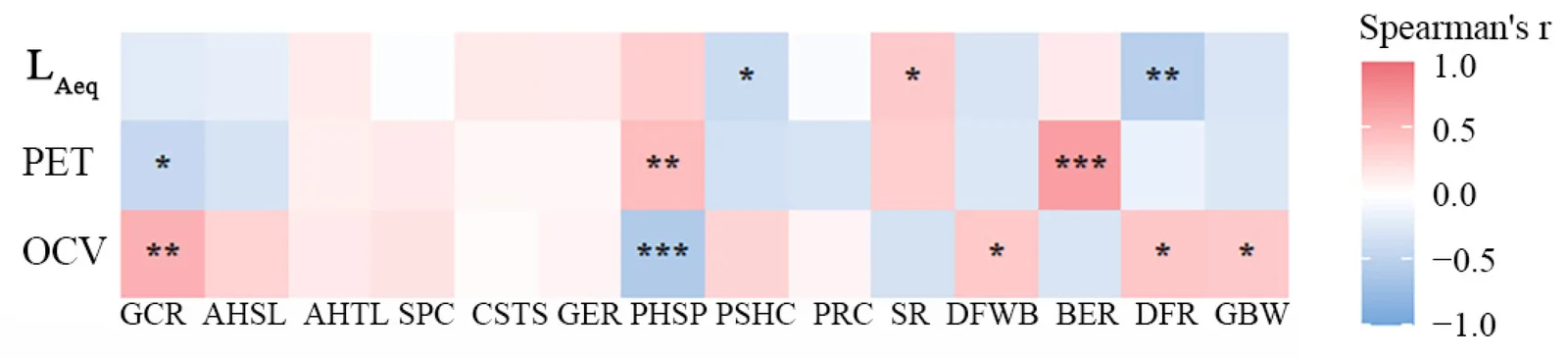
Correlation coefficients of key micro-landscape factors in the east–west orientation group. Note: * *p* < 0.05, ** *p* < 0.01, and *** *p* < 0.001.

In the east–west orientation group, green coverage rate, road distance, and green buffer width were positively correlated with OCV. Hard paving proportion was negatively correlated with OCV (*p* < 0.01). Green coverage rate was also negatively correlated with PET, whereas road distance was negatively correlated with L_Aeq_ (Table 8; Figure 6).

The correlation results showed orientation-dependent effects of landscape factors on OCV. Green coverage ratio, hard surface paving proportion, distance from the road, and greening buffer width showed consistent effect directions across the two orientation groups and were therefore identified as common key factors. Other variables, including tree crown spread, shrub-layer height, community structure, distance from water bodies, pavement properties, and building enclosure ratio, showed weaker or orientation-dependent associations and were therefore not included as universal predictors in the subsequent control regression models.

#### 2.4.2. Control Regression Analysis of Common Continuous Factors

Correlation analysis cannot distinguish the independent contribution of each factor when the factors act jointly. Therefore, controlled linear regression analysis was conducted using OCV as the dependent variable to estimate the average independent associations of the key continuous factors across the observed range. Green coverage rate and hard paving proportion showed a strong substitution relationship. Two separate models were therefore constructed (Models A and B).

Model A showed a significant overall fit (*R*^2^ = 0.668, adjusted *R*^2^ = 0.653, *p* < 0.001). Green coverage rate showed the strongest positive association with OCV (*β* = 0.612), followed by distance from the road (*β* = 0.476) and green buffer width (*β* = 0.306).

Model B, replacing green coverage with hard paving, was also significant (*R*^2^ = 0.684, adjusted *R*^2^ = 0.670, *p* < 0.001). Hard paving showed the strongest negative effect (*β* = −0.628), while road distance (*β* = 0.515) and green buffer width (*β* = 0.336) remained positively associated with OCV (Table 9).

**Figure 7:**
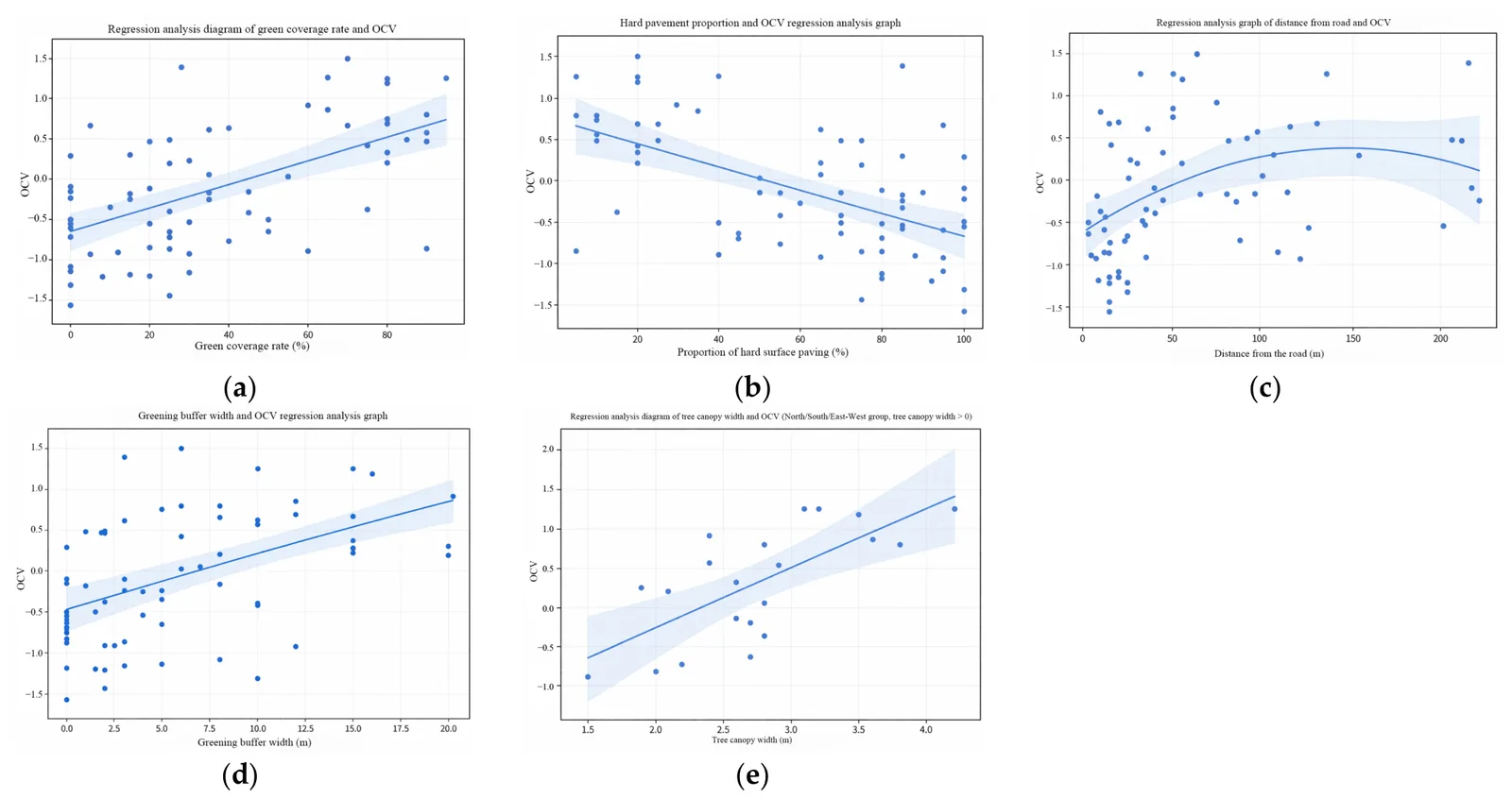
Single-factor response patterns between key landscape variables and overall comfort. (**a**) Regression Analysis Chart of Green Coverage Rate and OCV; (**b**) Regression Analysis Chart of Hard Surface Proportion and OCV; (**c**) Regression Analysis Chart of Distance from Road and OCV; (**d**) Regression Analysis Chart of Green Buffer Zone Width and OCV; (**e**) Regression Analysis Diagram of Tree Crown Spread and OCV.

Single-factor regression was used to explore the observed response patterns and identify favorable ranges rather than strict statistical thresholds (Figure 7). Unlike the controlled linear regression models, these single-factor relationships were used to describe the shape and distribution of OCV across individual landscape variables. The observed patterns showed diminishing associations for road distance and green-buffer width at higher values, indicating that the favorable ranges should not be interpreted as linear effect thresholds. Within the sampled sites, higher OCV was associated with approximately 40–55% green coverage and a hard paving ratio below 50%. Road distance showed a positive but diminishing association with OCV, with the increase becoming less pronounced at approximately 60 m and beyond. Green buffer width was positively associated with OCV, with relatively favorable levels observed within approximately 8–15 m. These ranges describe the observed response patterns and should be interpreted as context-specific design references rather than linear or universal thresholds. In north–south/northeast–southwest-oriented spaces, a tree canopy width of approximately 2.5–3.5 m was also associated with higher OCV. These values reflect the regression trends and observed distribution of the sampled sites and should therefore be interpreted as context-specific design references rather than universal thresholds. Their potential effects were further explored through numerical scenario simulations.

### 2.5. Model Evaluation and Scenario Assessment

The ENVI-met model was evaluated against field measurements at three representative monitoring points. During 08:00–18:00, the mean RMSE and MAPE were 0.33 °C and 0.91% for air temperature and 0.91% and 1.37% for relative humidity, respectively (Table 10). These results support the model’s ability to reproduce the measured air temperature and relative humidity conditions at the representative site. The evaluated model was subsequently used for scenario comparison. Because synchronized point-level measured and simulated PET data were not available, PET was treated as a scenario-response indicator rather than an independently validated metric.

For acoustic conditions, the Cadna/A model was used for comparative analysis of the 0, 5, and 8 m green buffer scenarios under consistent road traffic and spatial conditions. The mean L_Aeq_ reductions relative to the 0 m condition were 1.36 dB(A) for the 5 m buffer and 1.75 dB(A) for the 8 m buffer. These results were used to evaluate the relative acoustic effects of different buffer width scenarios rather than to independently validate the absolute noise levels of the model.

The ENVI-met scenario comparison indicated that the tested landscape configuration reduced PET and the spatial extent of high-temperature areas. These results provide scenario-based evidence for the potential thermal benefits of increased vegetation coverage and tree shading. The findings should be interpreted as simulation-based support rather than independent empirical validation of the proposed landscape ranges.

**Figure 8:**
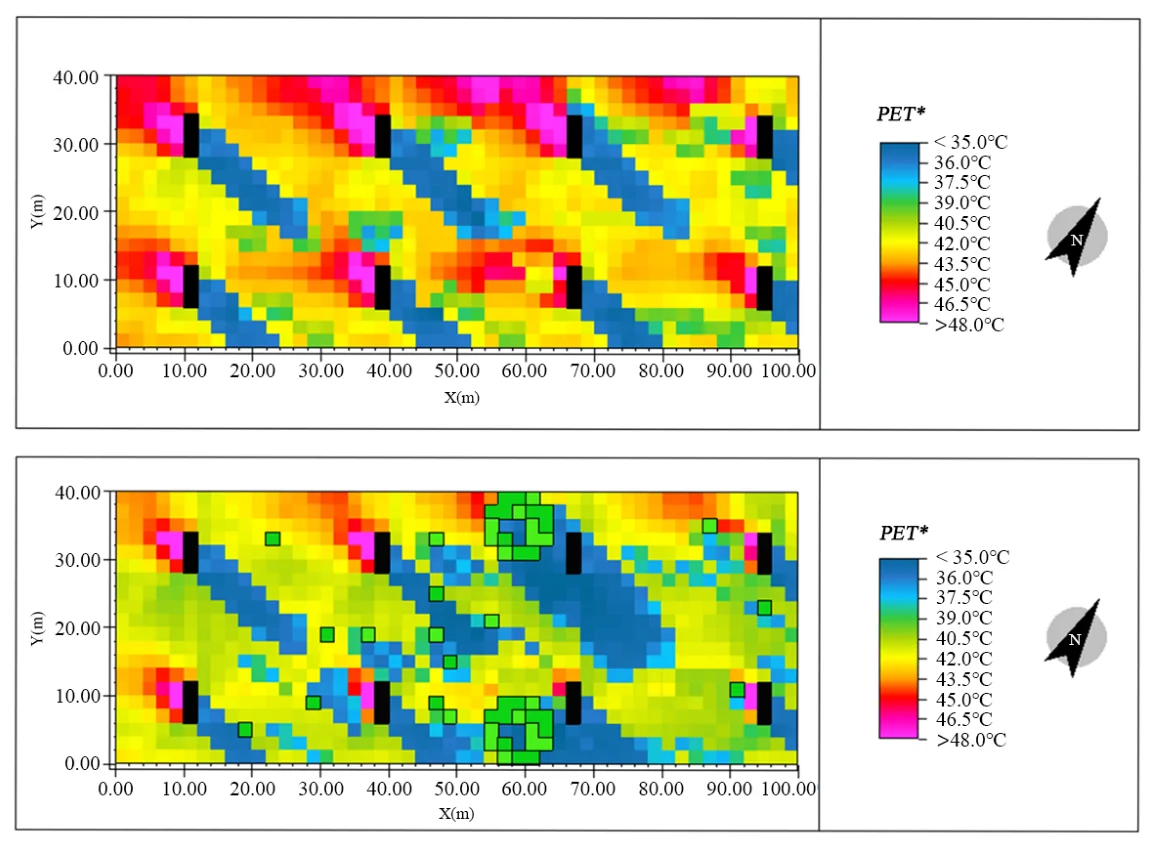
Comparison chart of PET Distribution between current operating conditions and optimized conditions. Note: * *p* < 0.05.

**Figure 9:**
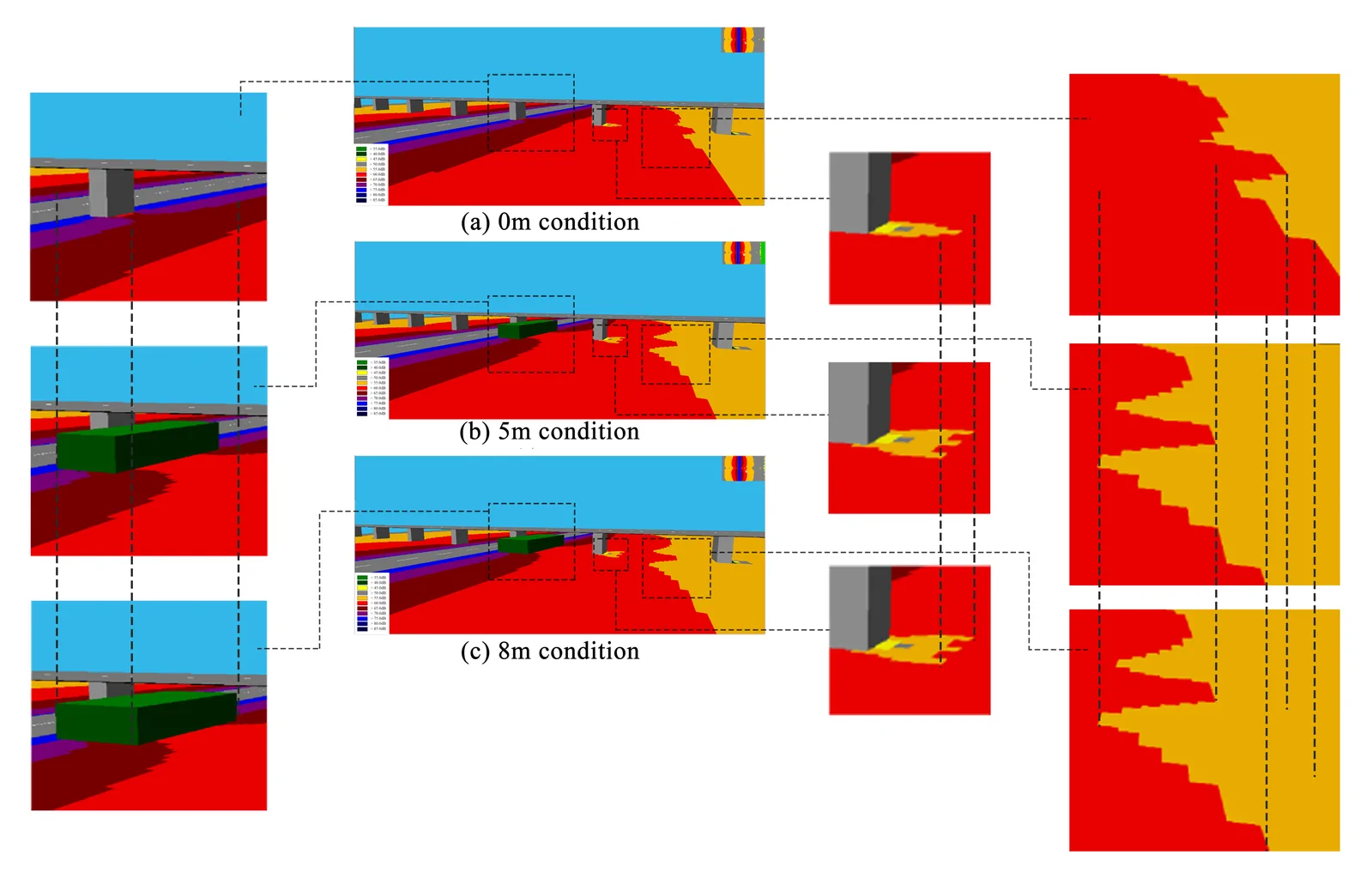
Three-dimensional perspective comparison diagram of daytime noise in the bridge underpass space under different conditions of green buffer zone width.

For acoustic conditions, the Cadna/A model was used to compare 0, 5, and 8 m green-buffer scenarios (Figure 8). The mean L_Aeq_ reductions relative to the 0 m condition were 1.36 dB(A) for the 5 m buffer and 1.75 dB(A) for the 8 m buffer. The results indicate that increasing the green buffer width can reduce traffic noise exposure, with the strongest effects occurring near the road boundary. As shown in Figure 9, as the width of the green buffer zone increases, the range of the high-noise area on the adjacent road gradually shrinks, and the relatively lower noise area inside the bridge is expanded. The scenario results suggest that increasing the green buffer width may reduce traffic noise exposure under the modeled conditions. The green buffer may therefore provide both landscape and potential acoustic benefits. These findings are intended to support scenario-based design comparison rather than to establish a universally applicable noise reduction threshold.

The simulation results indicated that the optimized landscape scenarios were associated with lower PET and more favorable acoustic conditions. Compared with the baseline scenario, the optimized scenarios showed more favorable thermal and acoustic conditions and higher simulated overall comfort. The simulation results were consistent with the statistical findings. They further supported the identification of the key landscape factors and their practical ranges. However, these ranges should be interpreted as practical design references rather than universal statistical thresholds.

## 3. Discussion

### 3.1. Contribution of Thermal Environment and Sound Environment to Overall Comfort

This study indicates that overall comfort in elevated bridge underpass spaces reflects the combined influence of thermal and acoustic environments, with thermal comfort showing a stronger association under the surveyed summer conditions (Table 11). This finding is consistent with previous studies showing that thermal and acoustic conditions jointly shape outdoor environmental perception [44,45]. In subtropical urban parks, vegetation can influence thermal and acoustic conditions through shading, evapotranspiration, and sound attenuation [46]. Jin et al. reported that these two dimensions explained 58.7–68.2% of overall comfort variation in urban squares [42], whereas the present study obtained an explanatory rate of 71.3%. The slightly higher explanatory performance may be related to the relatively persistent thermal and traffic noise exposure characteristic of elevated bridge underpass spaces. These effects can also influence overall environmental perception. Thermal comfort may further affect acoustic comfort [38]. The stronger contribution of thermal comfort may be related to the distinctive spatial conditions of elevated bridge underpass spaces. Overhead structures reduce direct solar exposure, but semi-enclosed boundaries and restricted airflow can limit heat dissipation and alter local radiation and humidity conditions. Vegetation can further modify these conditions through shading and evapotranspiration, while its spatial arrangement and distance from traffic sources may also influence sound propagation. Thus, plant configuration may simultaneously affect thermal exposure and acoustic exposure, providing a possible mechanism linking landscape characteristics with integrated environmental comfort [47,48,49].

Both PET and L_Aeq_ were significantly associated with overall comfort (*p* < 0.001), and their interaction was also significant (*p* = 0.013), indicating that the association between one environmental condition and OCV varied across levels of the other. At comparable L_Aeq_ levels, higher PET was associated with lower overall comfort and less favorable acoustic evaluations, whereas higher L_Aeq_ was associated with lower overall and thermal comfort at comparable PET levels. The reduced differences in OCV among thermal conditions at higher L_Aeq_ levels may indicate a masking effect between environmental stressors [50].

Two potential perceptual pathways may help explain this thermal–acoustic interaction. Traffic noise may increase psychological stress and environmental annoyance, thereby increasing sensitivity to concurrent thermal discomfort, while thermal stress and fatigue may reduce tolerance to traffic noise. However, physiological stress and psychological fatigue were not directly measured in this study. These pathways should therefore be regarded as plausible hypotheses for the observed cross-modal associations rather than confirmed causal mechanisms. Vegetation configuration may further influence both thermal and acoustic exposure through differences in radiation, air temperature and humidity, and sound propagation, thereby contributing to variations in overall comfort.

Therefore, improving overall comfort should not focus on a single environmental factor. Instead, plant landscape design should address thermal and acoustic conditions simultaneously. Multi-layer vegetation can increase canopy density, enhance evapotranspiration cooling, and reduce surface heat storage. These measures can improve the thermal environment. Green buffer zones can also reduce traffic noise exposure through absorption and scattering by vegetation. Within the sampled conditions, a green coverage rate of 40–55%, hard paving below 50%, and a green-buffer width of 8–15 m can be regarded as context-specific reference ranges rather than independent or fixed design thresholds. Their effects may depend on the combined vegetation configuration and surrounding spatial conditions.

### 3.2. Effects of Bridge Orientation and Spatial Form on Environmental Comfort

At the spatial form level, this study identified that the orientation of the bridge structure is the most distinct difference condition among the existing samples, while the regularity of the aspect ratio is relatively weak. Previous studies have pointed out that both the street valley orientation and the aspect ratio will have an impact on the thermal environment and thermal sensation of the pedestrian layer [51]. The orientation, aspect ratio, pavement material, and vegetation elements also jointly act on the thermal stress level [52]. The results of this study differ from some street valley studies, and the reason may lie in the continuous canopy cover feature of the elevated bridge underpass space, which is significantly different from the traditional urban street valley. The bridge structure reduces direct solar radiation entering elevated bridge underpass spaces. This effect may weaken the influence of aspect ratio on radiation exposure. However, bridge orientation still affects the lighting period, ventilation pathways, and plant growth conditions at the space edges. These differences can further influence under-viaduct microclimate formation. Therefore, bridge orientation should be regarded as an important spatial factor associated with differences in the environmental conditions of elevated bridge underpass spaces. From the perspective of spatial configuration, bridge orientation is associated with differences in shading patterns, light conditions, shadow distribution, and potential ventilation conditions, which may contribute to differences in the summer thermal environment. For regions with high summer temperatures and humidity such as Fuzhou and Xiamen, the microclimate differences formed by different bridge orientations are relatively obvious. East–west-oriented bridges can provide a relatively continuous shading range in summer. This configuration was associated with lower short-wave solar radiation entering elevated bridge underpass spaces. It may also lower surface and air temperatures and provide more stable conditions for shade-tolerant vegetation. Consequently, users may experience lower thermal exposure during hot periods. In contrast, the south–north-oriented and northeast–southwest-oriented elevated bridge underpass spaces are more susceptible to low-angle solar radiation, with significant changes in the light conditions at the edge area and significant changes in the shadow range over time, which is not conducive to the formation of a stable microclimate environment and increases the complexity of plant community configuration and maintenance management.

The orientation of the bridge structure should also take into account the prevailing wind conditions in summer. In the southeastern coastal areas, the prevailing winds in summer are mainly southeast and south winds. Therefore, when planning under-bridge spaces, the opening direction of the bridge structure should be considered together with the dominant wind direction to avoid obstructing natural ventilation. Good ventilation conditions can facilitate heat dissipation and air exchange, potentially contributing to a more favorable microclimate. However, the specific effects of plant configuration on airflow were not further simulated in this study and therefore require additional verification. Accordingly, vegetation planning should prioritize shading, thermal regulation, and adaptability to local environmental conditions, while the potential role of plant configuration in regulating airflow should be further investigated in future studies.

Therefore, bridge orientation can be regarded as an important spatial factor for consideration in the planning and renewal of elevated bridge underpass spaces, particularly because it determines the basic shading pattern and influences the light environment and potential ventilation pathways. Nevertheless, the observed orientation effects should be interpreted together with site-specific characteristics. Vegetation configuration, road distance, hard paving, and green buffer width may differ among individual sites and can also contribute to differences in environmental comfort. The control regression results further confirmed that several of these continuous landscape factors had independent associations with OCV, indicating that the observed comfort differences cannot be attributed to bridge orientation alone.

### 3.3. Plant Landscape Optimization Strategies for Elevated Bridge Underpass Spaces

At the micro-landscape level, the key factors identified in this study, such as green coverage rate and green buffer width, are generally consistent with the previous research conclusions on vegetation configuration and the environmental effects of road boundaries [53,54,55]. The results further confirm the role of vegetation in improving under-viaduct environmental conditions. Increasing green coverage and tree canopy size can reduce heat exposure through shading and evapotranspiration. Multi-layer vegetation may also reduce traffic noise interference and improve perceived environmental quality. These effects can jointly enhance thermal and acoustic comfort.

Green coverage rate is an important landscape indicator of under-viaduct microclimate and comfort. Previous studies suggest that shading and cooling effects become evident at approximately 30% green coverage and become stronger at 50–70%.

Due to the limitations of the bridge underpass space conditions, the effective green volume can be improved by increasing the multi-layer community of trees, shrubs, and grasses, as well as vertical greening and modular planting facilities. In Fujian region, climbing or hanging plants such as firecracker flower, large-flowered old duckbill, clematis, and Yunnan yellow hyacinth can be selected for planting, and vegetation should be prioritized around activity areas and pedestrian spaces which may contribute to more favorable microclimatic conditions.

**Figure 10:**
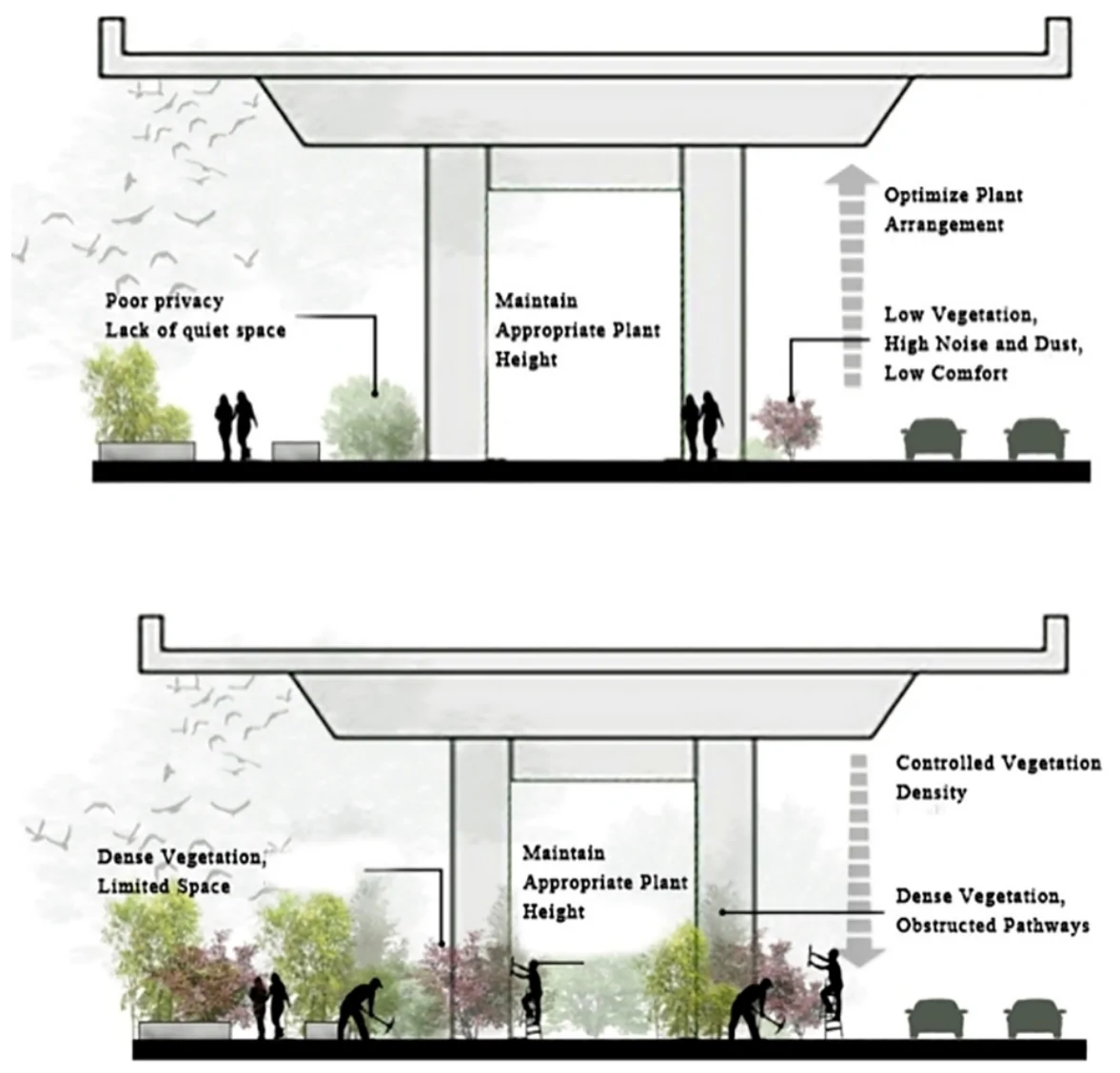
Recommended shrub-layer configuration based on the identified landscape factor.

Tree canopy size was particularly important in north–south and northeast–southwest-oriented spaces. In this study, a canopy width of 2.5–3.5 m was associated with better comfort. Continuous shade should therefore be prioritized around activity areas and along the bridge edges. At the same time, local tree species that are tolerant of partial shade, heat resistance and easy to maintain should be selected, such as hibiscus, cat-tail grass, small-leaf juniper, willow, large-flowered hibiscus, beech, heather, privet and magnolia, in order to enhance the ecological benefits of the vegetation. The average height of the shrub layer was identified as an important plant structural factor, particularly in the north–south and northeast–southwest-oriented spaces (Figure 10). During the optimization process, the configuration of the middle and lower layers of vegetation should be improved to enhance the continuity of the shrub layer; for dense areas, appropriate pruning and thinning should be carried out to maintain ventilation and spatial openness. Suitable shrub species that are tolerant to shade, heat, and moisture, such as octopus gold plant, splash gold coral, small-leaved, red leaf juniper, golden willow, southern bayberry, and magnolia, should be selected to enhance the stability of the plant community and its regulation ability of the bridge underpass microclimate. These landscape parameters should not be interpreted as independent design requirements. Their environmental effects may depend on the combined vegetation configuration, suggesting potential trade-offs among green coverage, green buffer width, canopy dimensions, and vertical vegetation structure. For example, where spatial constraints limit buffer width, increasing canopy coverage or developing a denser multi-layer vegetation structure may potentially compensate for part of the associated environmental function. However, the present study did not explicitly quantify interaction or substitution effects among these variables. Therefore, the identified ranges should be applied as context-specific references rather than interchangeable or fixed prescriptions.

**Figure 11:**
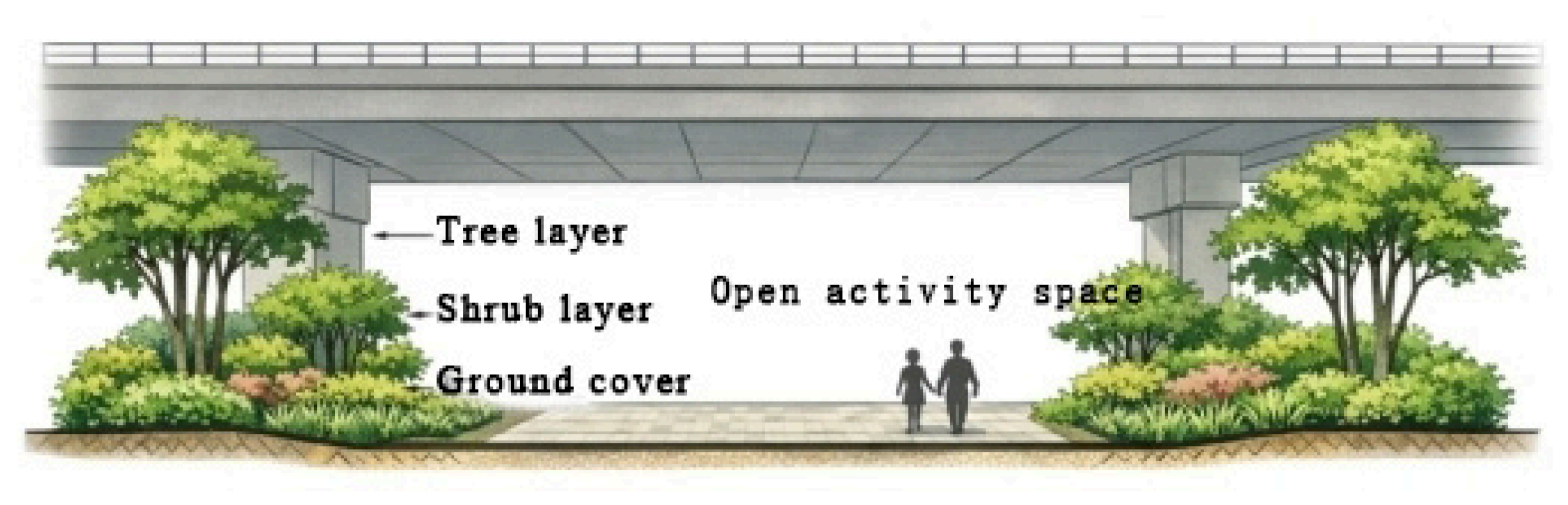
Conceptual multi-layer planting configuration based on the identified landscape factors.

Furthermore, compared with some studies that focus solely on the quantity of greenery, the results of this study indicate that the improvement in the comfort level of the under-bridge space is not only dependent on the increase in green coverage, but is also closely related to the plant configuration structure and the spatial organization method [56]. The community structure and green enclosure degree were identified as plant landscape factors associated with differences in microclimatic conditions. These associations were more pronounced in the north–south and northeast–southwest orientation groups. During the optimization process, a multi-layered plant community of trees, shrubs and grass should be constructed to enhance the completeness of the vegetation layers and the continuity of the green interface, in order to strengthen the shading, cooling and environmental buffering functions. The key configuration areas can include road boundaries, the surrounding of bridge piers and the periphery of activity spaces. By replanting shrubs and ground cover, integrating scattered green spaces, and establishing green buffer zones, the spatial coherence of the community can be improved while maintaining adequate space for user activities. Studies have shown that a multi-layered plant community of trees, shrubs and grass is more conducive to improving the microclimate and thermal comfort level of the under-bridge space in hot and humid areas. Therefore, the optimization of community structure should consider both microclimate regulation and spatial use requirements. Based on these findings, a multi-layer planting configuration of trees, shrubs, and grasses is proposed as a conceptual application of the identified landscape factors (Figure 11).

### 3.4. The Limitations of This Study and Suggestions for Future Research

This study has several limitations. First, the field investigation was conducted mainly during summer, and the findings therefore primarily reflect environmental exposure, comfort responses, and vegetation regulation under hot–humid summer conditions. Seasonal changes in vegetation growth, canopy structure, thermal exposure, and the relative importance of acoustic conditions were not captured [57]. In addition, the study included 12 elevated bridge underpass spaces in Fuzhou and Xiamen. Although both cities have a hot–humid subtropical climate, differences in urban morphology, traffic conditions, surrounding land use, and vegetation configuration may affect environmental exposure and comfort responses. Therefore, the identified relationships and landscape ranges should be regarded as context-specific empirical findings, and their transferability to other seasons, cities, and climatic regions requires further validation.

Second, multiple respondents were surveyed within each site, and the 1580 valid questionnaires therefore represent respondent-level observations rather than 1580 independent spatial samples. This hierarchical sampling structure should be considered when interpreting statistical significance and generalizability. Moreover, the observational design does not allow complete separation of bridge orientation from site-specific factors such as bridge height, traffic intensity, surrounding buildings, and shading duration, and self-reported comfort evaluations may involve respondent selection and perception bias. Although subgroup analyses showed generally consistent associations among TCV, ACV, and OCV across gender, age, and outdoor stay duration groups, they do not fully capture individual-level heterogeneity or thermal adaptation. Similarly, the proposed thermal–acoustic pathways were derived from correlation and regression evidence. Mediation and structural equation analyses were not conducted, and the directionality and indirect effects among environmental exposure, sensation, comfort, and overall comfort therefore require further statistical verification.

Third, limitations remain in the numerical simulations and the interpretation of the proposed landscape references. The Cadna/A simulation was not quantitatively validated against field measurements because corresponding point-level measured and simulated L_Aeq_ data were unavailable; therefore, its results should be interpreted as relative comparisons among buffer-width scenarios rather than validated absolute noise predictions. In addition, simplification of vegetation parameters and boundary conditions may affect simulation accuracy. The identified landscape ranges, including green coverage and green-buffer width, should not be interpreted as independent or universally applicable thresholds. Potential interaction, trade-off, and substitution effects among green coverage, buffer width, canopy dimensions, vertical vegetation structure, and plant species were not explicitly quantified. Accordingly, the planting configurations presented in Figure 10 and Figure 11 should be regarded as conceptual applications of the empirical findings rather than independently validated design scenarios.

Future studies should extend field monitoring across seasons, cities, and climatic regions and adopt hierarchical or mixed-effects statistical approaches to better account for the nested structure of respondents and sites. Independent acoustic validation and additional scenario testing are also needed to improve the robustness of simulation-based design recommendations. Further research could quantify interactions and potential substitution effects among landscape variables and incorporate physiological indicators, behavioral observations, and sensor-based tracking to examine the proposed thermal–acoustic perceptual pathways. Integrating these approaches with digital-twin-based simulation may provide a more comprehensive framework for evaluating and optimizing elevated bridge underpass spaces.

## 4. Materials and Methods

**Figure 12:**
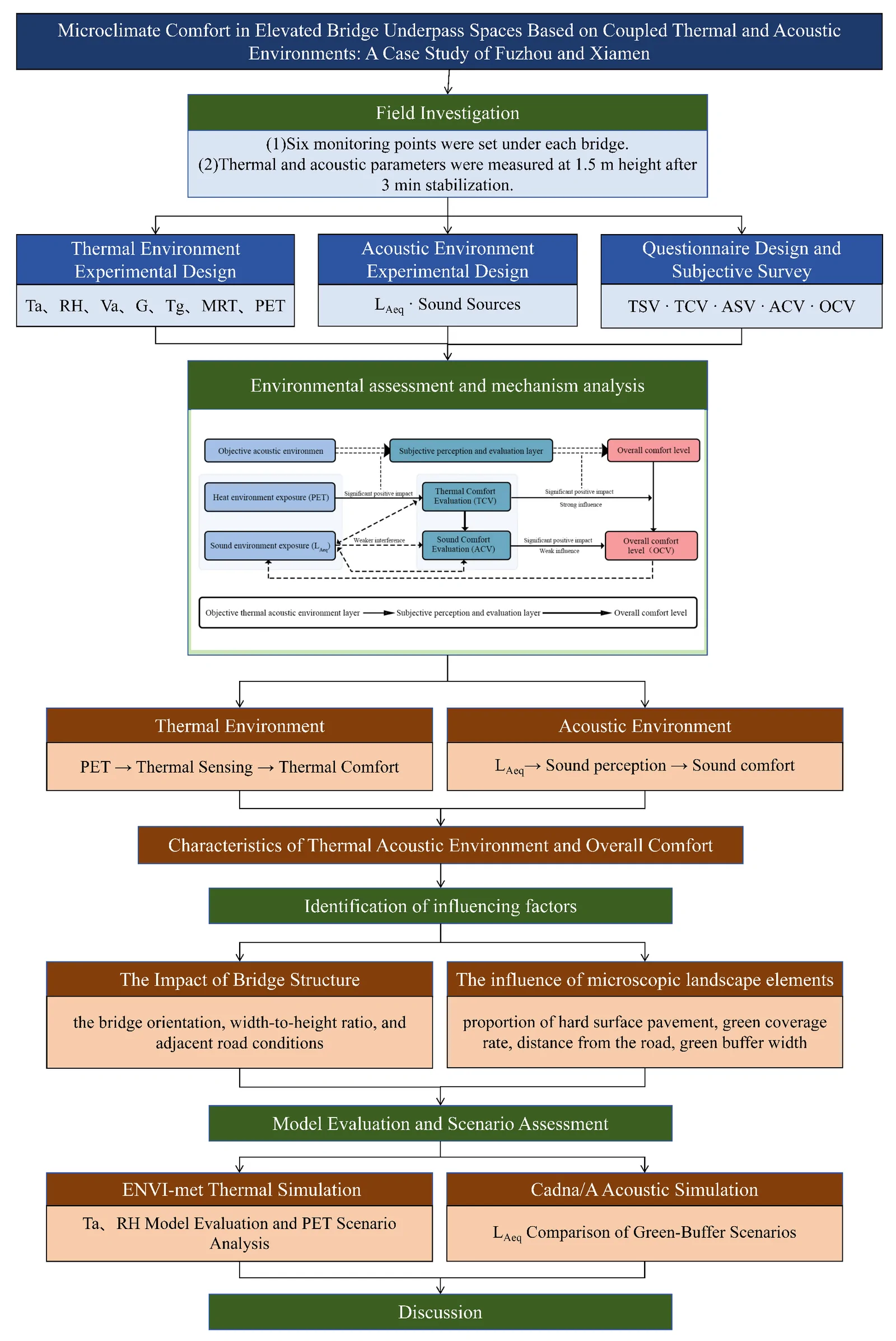
Technology roadmap.

To systematically investigate the determinants of urban microclimate and environmental comfort in elevated bridge underpass spaces in hot–humid regions, this study establishes a technical framework comprising “field investigation–mechanism identification–simulation-based scenario assessment” (Figure 12). The framework consists of three stages. The first stage involves on-site monitoring of thermal and acoustic environments and subjective questionnaire surveys. The second stage involves statistical and quantitative analyses of macro- and micro-scale environmental factors. The third stage involves optimization strategies evaluated through numerical scenario analysis. During this process, software tools such as RayMan (Version 3.1 Beta), ENVI-met (Version 5.9.0), and Cadna/A (Version 2026 MR1) are primarily used for indicator calculation and environmental simulation, while SPSS (Version 27.0) is employed for statistical analysis, ultimately generating optimized parameters and zoning management outcomes for bridge underpass spaces.

### 4.1. Selection of Study Areas

**Figure 13:**
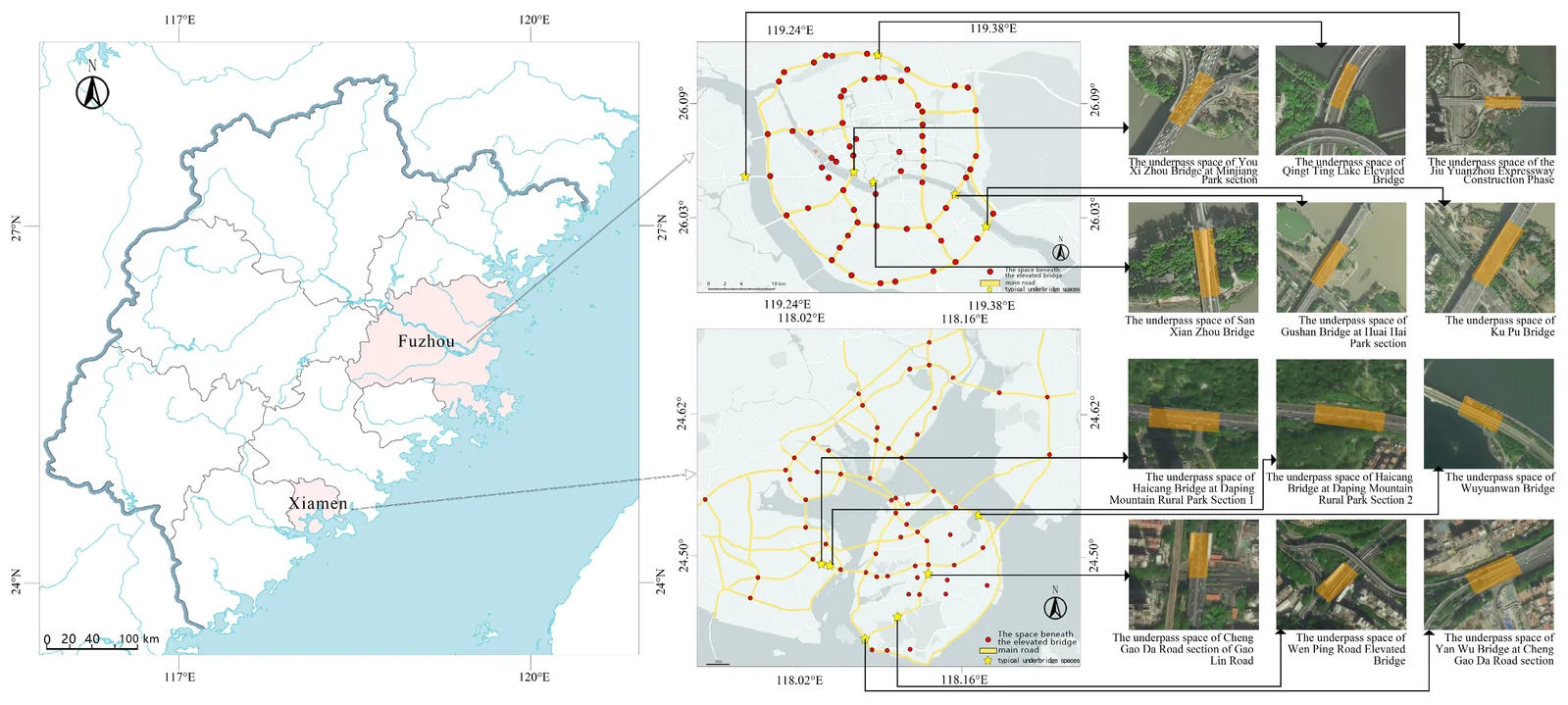
Distribution map of elevated bridge underpass spaces in Fuzhou and Xiamen.

This study selected Fuzhou and Xiamen in Fujian Province as the study areas because both cities have a typical hot–humid climate and diverse elevated transportation infrastructure. Based on a preliminary census, candidate elevated bridge underpass spaces were screened according to their stable public use, recreational and social functions, accessibility, diversity in spatial and traffic conditions, and suitability for field monitoring. Twelve representative elevated bridge underpass activity spaces were finally selected, including six in Fuzhou and six in Xiamen elevated bridge underpass spaces (Figure 13). During site selection, major differences in vegetation, road exposure, and spatial enclosure were considered to reduce potential confounding among the sampled sites.

### 4.2. Research Design

To investigate the microclimatic characteristics and environmental impacts of elevated bridge underpass activity spaces, this study conducted field monitoring and subjective questionnaire surveys in Fuzhou and Xiamen, two hot–humid subtropical cities in southeastern China. Objective data on thermal environment, acoustic environment, and landscape features are collected simultaneously, complemented by user perception evaluations, providing data support for identifying the influence mechanisms of landscape elements at different scales on environmental comfort.

#### 4.2.1. Thermal Environment Experimental Design

To characterize the summer thermal environment of bridge underpass activity spaces, this study employs HOBO miniature weather station recording systems and JT2011 black globe thermometers for field monitoring. The measured parameters included air temperature (T_a_), relative humidity (RH), wind speed (V_a_), solar radiation intensity (G), and globe temperature (T_g_). These variables characterized the microclimatic and radiant conditions of the underpass spaces. They were also used to calculate mean radiant temperature (MRT) and PET. Air temperature and relative humidity were measured using a HOBO S-THC-M002 sensor, wind speed using a HOBO S-WCF-M003 sensor, and solar radiation using a HOBO S-LIB-M003 sensor (Onset Computer Corporation, Bourne, MA, USA). Globe temperature was measured using a JT2011 black globe thermometer (Beijing Century Jiantong Technology Co., Ltd., Beijing, China). Technical specifications and measurement accuracies of each instrument are shown in Table 12. To ensure data quality, all instruments were calibrated before formal monitoring and operated according to standardized procedures. The measurement accuracies reported in Table 1 were used as instrument-level uncertainty ranges for the monitored variables.

#### 4.2.2. Acoustic Environment Experimental Design

Acoustic environment monitoring is conducted using the BSWA309 multifunctional sound level meter (BSWA Technology Co., Ltd., Beijing, China), adopting L_Aeq_ as the evaluation index according to the “Environmental quality standard for noise” (GB 3096-2008) [58]. Monitoring was carried out from 08:00 to 18:00, with continuous sampling at 1 s intervals, and each measurement point was monitored continuously for 5 min, with L_Aeq_ representing the acoustic environment characteristics. During monitoring, instruments were uniformly calibrated and equipped with windshields, and major noise sources—including traffic noise, human activities, and natural sounds—were recorded to support the analysis of the coupled response between microclimate and environmental comfort in bridge underpass spaces.

#### 4.2.3. Measurement Point Layout and Field Monitoring Methods

**Figure 14:**
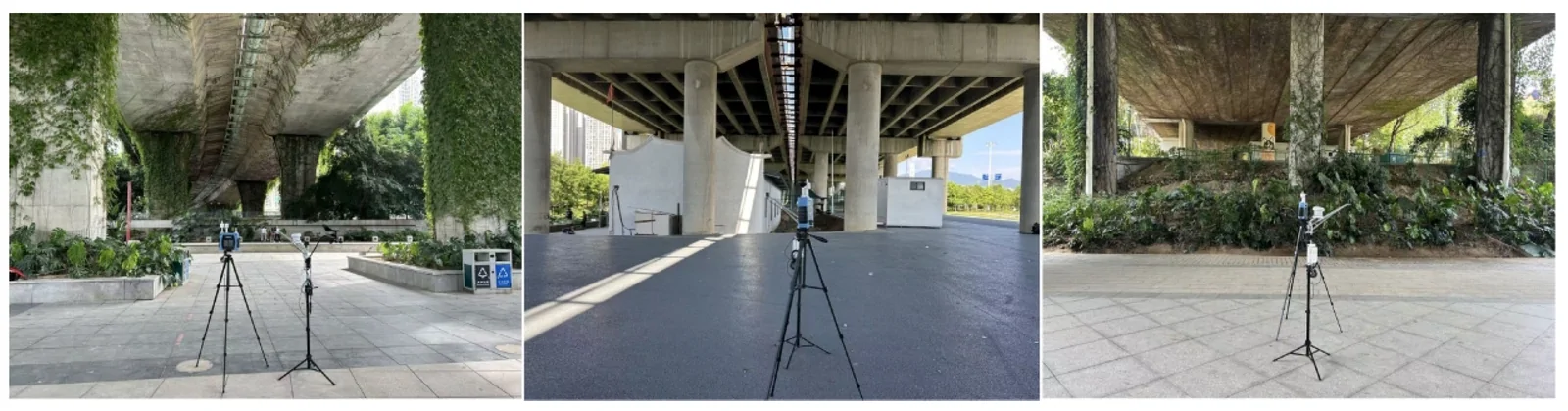
Representative monitoring point configurations in elevated bridge underpass activity spaces, with equipment placed at a height of 1.5 m.

The measurement area was defined as the relatively complete space between two bridge piers and within the bridge projection range. Six fixed points were arranged in each site considering road influence, human activity distribution, vegetation conditions, and spatial openness, covering both disturbed and relatively stable areas to improve spatial representativeness and inter-site comparability (Figures S1–S12). Figure 14 shows three representative monitoring point configurations. Thermal and acoustic parameters were measured simultaneously. The monitoring height was set at 1.5 m above ground level, corresponding approximately to the human breathing zone. To minimize the impact of transient disturbances caused during instrument movement and setup on measurement results, a stabilization period of at least 3 min was implemented at each site prior to formal data collection. During field surveys, temporary shading, water sprinkling for cooling, crowd gatherings, and traffic incidents were also recorded as anomalies to support subsequent data verification and quality control.

**Figure 15:**
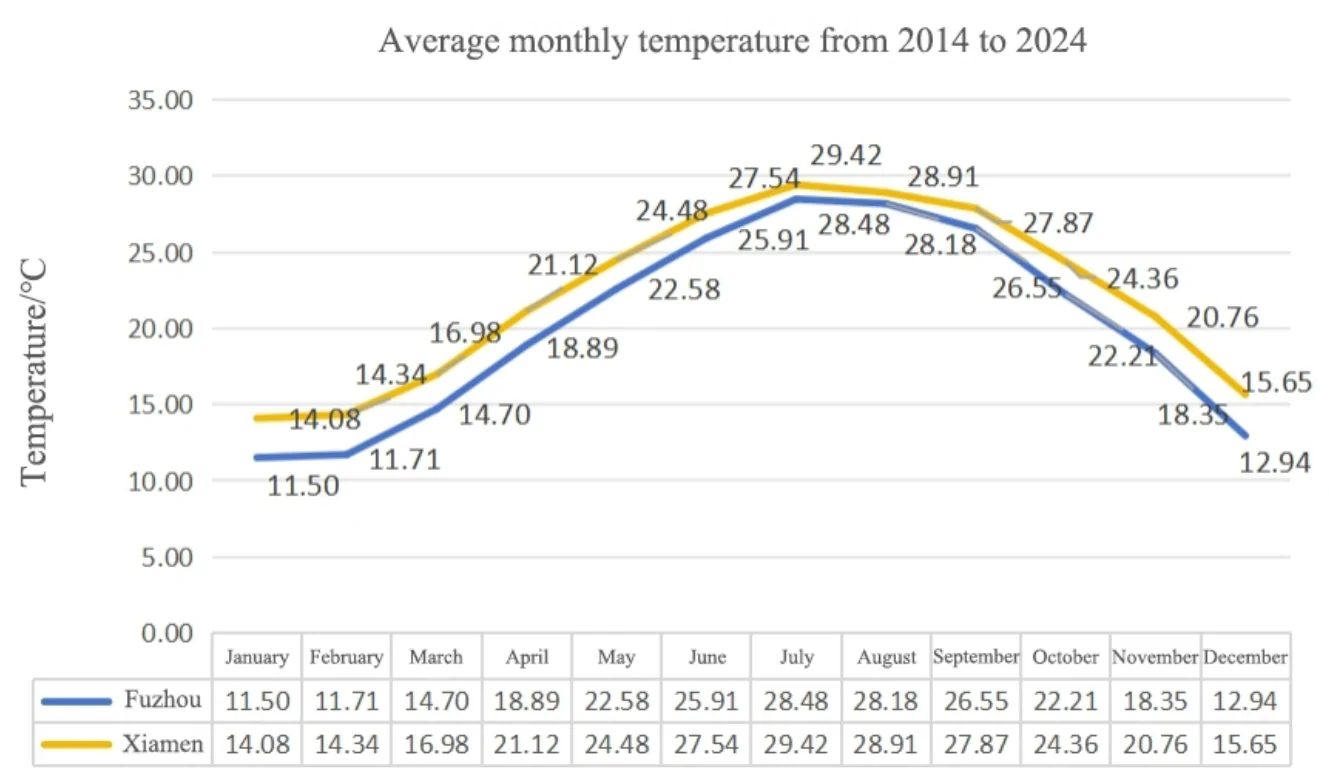
Monthly average temperature changes in Fuzhou and Xiamen over the past 10 years.

Fuzhou and Xiamen have a typical hot–humid subtropical climate, with high annual temperatures and abundant precipitation. Based on long-term meteorological records, July and August are the hottest months of the year (Figure 15); therefore, field measurements and surveys were conducted during summer under relatively stable weather conditions. The 12 elevated bridge underpass spaces were measured on 1–3 July and 8–10, 21–22, and 24–27 August 2025. Rainy and extreme weather days were excluded, and measurements were conducted under clear, mostly cloudy, or cloudy conditions with light winds. The specific measurement dates and weather conditions for each site are summarized in Table 13. All measurements were conducted during the summer period under comparable hot–humid climatic conditions, ensuring seasonal consistency across the field campaigns.

#### 4.2.4. Questionnaire Design and Subjective Survey

To obtain subjective evaluations from users of elevated bridge underpass activity spaces regarding thermal environment, acoustic environment, and overall environmental quality, this study conducted a questionnaire survey concurrently with field measurements. A total of 1618 questionnaires were collected, of which 1580 were valid. The 1580 valid questionnaires represent respondent-level observations collected across the 12 selected physical sites. They therefore reflect repeated user evaluations within the same spatial settings rather than 1580 independent physical site samples. The questionnaire consisted of two parts: first, basic information about respondents (e.g., clothing and activity status), these individual characteristics were retained for subsequent sensitivity and subgroup analyses to examine whether demographic and habitual use differences affected comfort responses. The second part consisted of subjective perception assessments, including Thermal Sensation Vote (TSV), Acoustic Sensation Vote (ASV), Thermal Comfort Vote (TCV), Acoustic Comfort Vote (ACV), Thermal Preference Vote (TPV), Acoustic Preference Vote (APV), and Overall Comfort Vote (OCV). The OCV represents the overall subjective evaluation of environmental comfort in the elevated bridge underpass space under the combined influence of thermal and acoustic conditions. A seven-point Likert scale was used; however, for TSV, it was expanded to a nine-point scale to better capture extreme perceptual differences, given summer thermal conditions. The numerical scores corresponded to ordered verbal categories. Negative and positive values represented opposite perceptual directions, while 0 represented a neutral or moderate response. Evaluation content and scoring methods for each indicator are detailed in Table 14. The questionnaire also collected respondents’ subjective acceptance levels, preferred changes, and desired improvements regarding environmental factors such as solar radiation, temperature, humidity, and wind speed, providing reference data for analyzing relationships between subjective perceptions and environmental characteristics.

To ensure data quality, reliability and validity, tests were conducted on the questionnaire. Results showed a Cronbach’s *α* coefficient of 0.794, indicating good internal consistency; the KMO value was 0.812, and Bartlett’s sphericity test reached significance (*p* < 0.001), suggesting that the data were suitable for factor analysis. Factor analysis revealed that cumulative variance explained by common factors reached 81.54%, and factor loadings for all items were at acceptable levels, confirming strong structural validity. Therefore, the questionnaire provides a reliable data foundation for evaluating thermal comfort, acoustic comfort, and overall comfort in elevated bridge underpass activity spaces.

### 4.3. Selection of Environmental Parameters for Integrating Objective and Subjective Assessments of Thermal and Acoustic Environments

Based on established thermal comfort assessment methods, this study selected the Physiologically Equivalent Temperature (PET) as the primary thermal indicator [13]. PET values were calculated using the RayMan Pro (Version 3.1 Beta) model based on air temperature, relative humidity, wind speed, mean radiant temperature, clothing insulation, and metabolic rate [59]. Mean radiant temperature was calculated following the ISO 7726 [60] methodology based on measured globe temperature, air temperature, wind speed, and solar radiation, and was subsequently used as an input to the RayMan model. The calculated PET values were matched with the subjective responses according to the questionnaire completion time.

This approach enables the establishment of correlations between individual thermal exposure levels and subjective perceptions of thermal and acoustic comfort, as well as overall comfort. PET provides a comprehensive representation of human thermal exposure and thermal perception in outdoor environments. Therefore, PET was selected as the core indicator of thermal conditions. It was used in subsequent correlation analyses and in the assessment of thermal–acoustic interactions. The relationships between PET, other thermal parameters, and subjective evaluations were also examined. These parameters included air temperature, mean radiant temperature, wind speed, and relative humidity. The analysis was used to identify the parameters most representative of thermal perception in elevated bridge underpass spaces. Since there is currently no established comprehensive parameter for assessing acoustic environment perception, L_Aeq_ is adopted as the single indicator to reflect the acoustic environment beneath bridges.

### 4.4. Development of the Overall Comfort Evaluation Model

**Figure 16:**
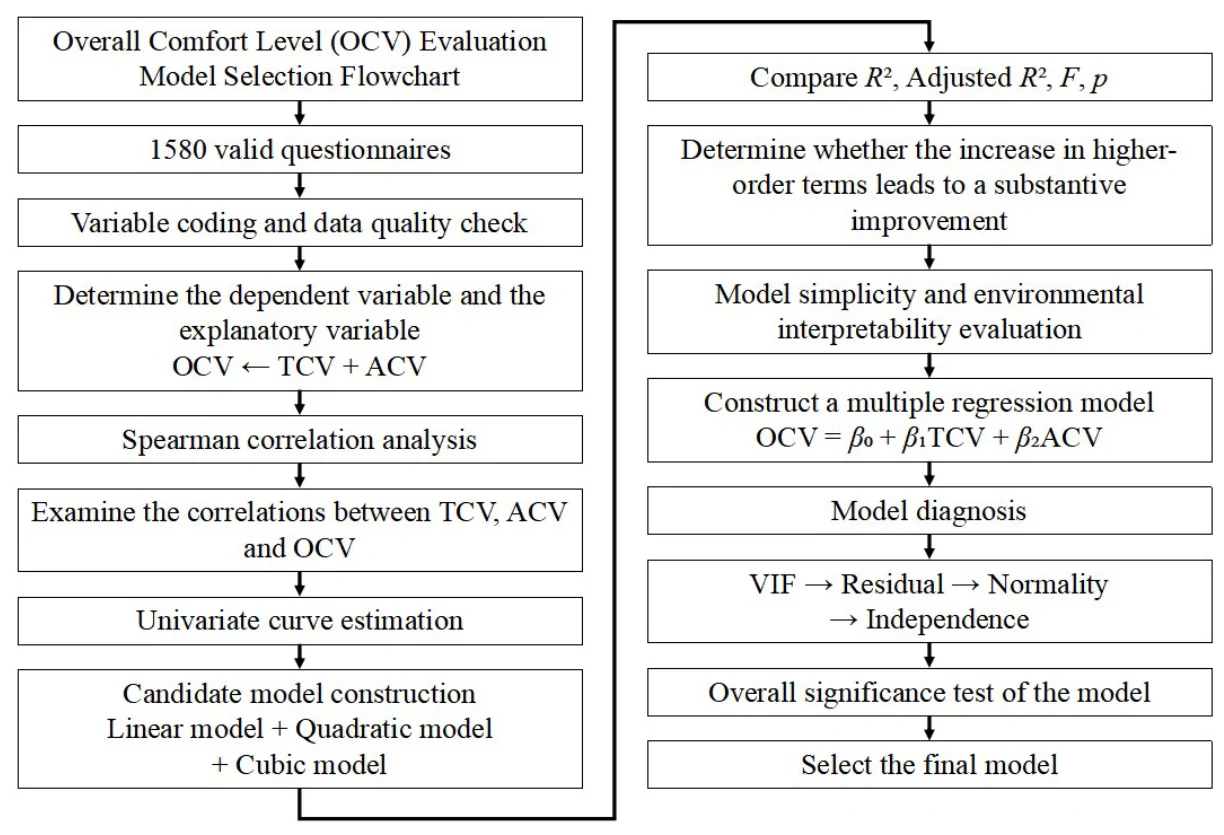
Technical roadmap of the overall comfort evaluation model.

To quantitatively investigate the combined effects of the thermal and acoustic environments on overall environmental comfort in elevated bridge underpass spaces, regression analysis was conducted based on 1580 valid questionnaires. OCV was specified as the dependent variable, while TCV and ACV were selected as the core explanatory variables. Linear, quadratic, and cubic models were first compared based on statistical significance and goodness of fit. Multivariate regression was then performed. Model diagnosis and final model selection followed. This stepwise procedure provided a statistically justified and reproducible framework for constructing the overall comfort evaluation model (Figure 16).

#### 4.4.1. Variable Determination and Data Preprocessing

First, the questionnaire data were uniformly coded, and all subjective evaluation indicators were converted into numerical variables according to the rating scales presented in Table 14. TCV, ACV, and OCV were all assessed using seven-point scales ranging from −3 to 3. As these indicators represent perceptual changes from negative to positive states, their original coding directions were retained in the regression analysis; higher values of TCV, ACV, and OCV indicate higher levels of thermal comfort, acoustic comfort, and overall comfort, respectively.

OCV was specified as the dependent variable:

*Y* = OCV (1)

TCV and ACV were specified as the two core explanatory variables:

*X*_1_ = TCV; *X*_2_ = ACV (2)

Descriptive statistics were first calculated for all variables. For the candidate landscape predictors, Spearman rank correlation analysis was used to examine their interrelationships and potential redundancy before model development. The associations among TCV, ACV, and OCV were also examined to assess the preliminary relationships among the core comfort variables. Spearman rank correlation was selected for pairwise association analysis because TCV, ACV, and OCV are seven-point ordered responses, and the analysis aimed to assess the strength and direction of monotonic associations rather than estimate an ordinal outcome model. Ordinal regression is more appropriate when the primary objective is to model an ordered dependent variable and estimate the effects of multiple predictors; therefore, it was not used for the preliminary pairwise correlation analysis. These correlation analyses were used primarily for preliminary assessment of variable relationships and were not used as the sole criterion for selecting variables for the multivariate regression model. Landscape variables showing consistent associations with OCV across bridge-orientation groups were retained as candidate predictors, while variables with weaker or orientation-dependent associations were considered separately. Potential multicollinearity among the retained predictors was subsequently assessed using VIF. Given that TCV and ACV represent two theoretically distinct and environmentally meaningful dimensions of comfort, both variables were retained as core explanatory variables in the subsequent overall comfort regression model. Both TCV and ACV were retained as core explanatory variables in the subsequent overall comfort regression model, and VIF [61] was used to assess potential multicollinearity:

(3) $${\mathrm{VIF}}_{i}=\frac{1}{1\;-\;R_{i}^{2}}$$

where $R_{i}^{2}$ is the coefficient of determination obtained from an auxiliary regression in which the i-th explanatory variable is treated as the dependent variable and the remaining explanatory variables are used as predictors. A VIF value below 5 is commonly considered indicative of no substantial multicollinearity, whereas a VIF of 10 or greater generally indicates severe multicollinearity.

#### 4.4.2. Candidate Functional Forms and Model Specification

Overall comfort may exhibit different degrees of linearity or nonlinearity. The curve estimation procedure in SPSS 27.0 was first employed to examine the functional relationships of TCV and ACV with OCV, separately.

For TCV and ACV, linear, quadratic, and cubic regression models were separately fitted. The linear model was expressed as:

OCV = *β*_0_ + *β*_1_X + *ε* (4)

The quadratic model was expressed as:

OCV = *β*_0_ + *β*_1_X + *β*_2_X^2^ + *ε* (5)

where X represents either TCV or ACV; *β*_0_ is the intercept; *β*_1_, *β*_2_, and *β*_3_ are the regression coefficients to be estimated; and *ε* denotes the random error term.

The original TCV and ACV variables are ordinal-scale measures containing negative, zero, and positive values. Direct logarithmic transformation of these variables is mathematically undefined for non-positive values. Therefore, logarithmic functional forms based directly on the original TCV and ACV values were not considered in this study.

#### 4.4.3. Model Selection Criteria

To avoid determining the final model form based on a single statistical criterion, a four-level model selection framework incorporating statistical significance, goodness of fit, model complexity, and interpretability was established.

The overall significance of each candidate regression model was evaluated using the F-test. A model was considered to have statistically significant explanatory power when the overall test reached the significance level of *p* < 0.05; otherwise, it was excluded from further consideration as a final candidate model.

The *F*-statistic [62] was calculated as:

(6) F=SSRkSSEn − k − 1

where SSR is the regression sum of squares, SSE is the residual sum of squares, *n* is the effective sample size, and *k* is the number of explanatory variables or effective regression terms in the model.

For candidate models that achieved statistical significance, the coefficient of determination (*R*^2^) [63] and adjusted coefficient of determination (Adjusted *R*^2^) [63] were further compared. *R*^2^ was used to evaluate the explanatory power of each model, whereas adjusted *R*^2^ was considered to account for the increase in model complexity associated with additional polynomial terms. Because the primary objective of this model was to obtain an interpretable relationship between subjective thermal–acoustic comfort and overall comfort rather than to maximize predictive performance, model complexity was considered together with goodness of fit and diagnostic results. Therefore, higher-order terms were retained only when they provided a meaningful improvement in explanatory performance and a stable, interpretable relationship. They were calculated as:

(7) $$R^{2}=1\;-\;\frac{\mathrm{SSE}}{\mathrm{SST}}$$

(8) Adjusted R2=1 − 1 − R2n − 1N − k − 1

where SST is the total sum of squares. A higher *R*^2^ indicates a greater ability of the model to explain variations in overall comfort. Adjusted *R*^2^ further accounts for the number of model parameters and was therefore used to assess whether the inclusion of higher-order terms resulted in a meaningful improvement in explanatory power.

For quadratic and cubic models, higher-order models were not preferentially selected when the inclusion of additional terms resulted only in limited increases in *R*^2^ or adjusted *R*^2^. In particular, when the addition of higher-order terms did not produce a substantial improvement in model fit, the principle of parsimony was followed to avoid overfitting.

Accordingly, an increase in model complexity was considered justified only when it resulted in a substantial improvement in model fit, rather than merely a marginal increase in *R*^2^.

The final model was required not only to demonstrate satisfactory statistical performance but also to have clear environmental interpretability and a relatively small number of parameters. When different models exhibited comparable fitting performance, the model with a simpler structure, fewer parameters, and greater interpretability was preferred.

Overall, the final functional form was associated according to the following sequence: statistical significance → higher goodness of fit → substantial improvement resulting from additional parameters → better interpretability.

#### 4.4.4. Univariate Model Selection

Based on the above criteria, the linear, quadratic, and cubic models describing the relationships between TCV/ACV and OCV were compared separately (Table 15). For TCV, the *R*^2^ increased from 0.578 for the linear model to 0.596 after the quadratic term was introduced, representing an increase of 0.018. With the further addition of a cubic term, *R*^2^ increased to 0.603, corresponding to a cumulative increase of 0.025 relative to the linear model. For ACV, *R*^2^ increased from 0.292 for the linear model to 0.308 for the quadratic model and further to 0.314 for the cubic model, representing a cumulative increase of 0.022 relative to the linear model.

These results indicate that although the higher-order models provided statistically higher explanatory power, the incremental improvement was relatively limited. For TCV, the increase in *R*^2^ from the linear to cubic model was only 0.025, while for ACV the cumulative increase was 0.022. Thus, the higher-order terms accounted for only a small additional proportion of the variance. Considering that quadratic and cubic specifications introduce additional parameters and may capture sample-specific curvature without providing a substantial improvement in explanatory performance, the higher-order models were not selected solely on the basis of their higher *R*^2^ values. Instead, the linear specification was retained as a more parsimonious and interpretable representation of the dominant relationship between subjective thermal/acoustic comfort and overall comfort.

#### 4.4.5. Construction of the Multivariate Overall Comfort Regression Model

Because overall environmental comfort is simultaneously affected by thermal and acoustic conditions, the univariate curve models can only characterize the individual relationship between TCV or ACV and OCV and cannot adequately describe their combined effects. Therefore, based on the univariate model analysis, TCV and ACV were simultaneously incorporated into a multivariate regression model.

First, a multivariate linear model was established as follows [64]:

OCV = *β*_0_ + *β*_1_TCV + *β*_2_ACV + *ε* (9)

where *β*_0_ is the regression intercept, and *β*_1_ and *β*_2_ represent the independent effects of TCV and ACV on OCV, respectively, while controlling for the other explanatory variable.

The overall significance of the regression model was first evaluated using the F-test, while the *t*-tests of the regression coefficients were used to associate whether each explanatory variable had a statistically significant independent contribution. The regression model was developed to examine the associations between thermal perception, acoustic perception, and overall comfort. It was not intended to establish causal relationships among environmental variables. The overall explanatory performance of the model was evaluated using *R*^2^ and adjusted *R*^2^.

For the overall comfort model containing two explanatory variables, the overall *F*-statistic was calculated as:

(10) F=R2k1 − R2n − k − 1

With 1580 valid respondent-level observations and *R*^2^ = 0.713, the overall *F*-statistic was calculated as:

F=0.71321 − 0.7131580 − 3≈1961.1

Here, the 1580 valid questionnaires represent respondent-level observations collected across the 12 selected physical sites, rather than 1580 independent spatial samples. The overall model was statistically significant (*p* < 0.001). The adjusted coefficient of determination was calculated as:

Adjusted R2=1 − 1 − 0.7131580 − 11580 − 3≈0.7126

This indicates that, after accounting for the number of model parameters, the model still explained approximately 71.3% of the variance in overall comfort. To further examine the interaction between thermal and acoustic conditions, a two-way ANOVA was conducted with OCV as the dependent variable and PET and L_Aeq_ as the two factors. The analysis examined the main effects of PET and L_Aeq_ and their interaction effect on OCV. Statistical significance was evaluated at *p* < 0.05.

#### 4.4.6. Model Diagnosis and Robustness Assessment

After determining the overall comfort regression model, basic model diagnostics were performed. First, the variance inflation factor (VIF) was used to assess multicollinearity among the explanatory variables, with VIF < 5 considered indicative of no substantial multicollinearity. Second, standardized residual plots and Q–Q plots were examined. The residual plots were used to assess linearity and homoscedasticity. The Q–Q plots were used to assess the normality of the residual distribution. A model was considered to reasonably satisfy the major assumptions of linear regression when the residuals were randomly distributed around zero and generally followed the expected theoretical distribution. In addition, potential outliers and influential observations were examined as supplementary diagnostics to minimize the potential influence of a small number of extreme observations on the regression results. The final model was then evaluated in terms of its applicability and stability based on the overall diagnostic results. These diagnostic checks were used to verify whether the selected linear specification adequately satisfied the main regression assumptions and whether the observed improvement of higher-order models was necessary from a model-diagnostic perspective. The diagnostic results did not indicate a clear violation of the linear model assumptions or a strong need for additional polynomial terms.

#### 4.4.7. Final Model Determination

Based on the combined results of the univariate curve estimation and multivariate regression analyses, both TCV and ACV showed significant associations with OCV, and their simultaneous inclusion in the model provided a satisfactory explanation of variations in overall comfort. Although the quadratic and cubic models yielded slightly higher *R*^2^ values than the linear model, the incremental explanatory power provided by the higher-order terms was limited. In addition, the residual and Q–Q plot diagnostics of the final linear model did not indicate substantial violations of the major regression assumptions. Therefore, considering statistical significance, goodness of fit, adjusted explanatory power, parameter parsimony, diagnostic performance, model stability, and environmental interpretability, the multivariate linear regression model was ultimately selected as the overall comfort evaluation model for elevated bridge underpass spaces.

The final model was expressed as:

OCV = 0.12 + 0.68TCV + 0.39ACV(11)

The coefficient of determination was:

*R*^2^ = 0.713

and the adjusted coefficient of determination was:

Adjusted *R*^2^ = 0.713

The overall model test yielded:

*F* = 1958.89, *p* < 0.001

These results indicate that TCV and ACV jointly explained approximately 71.3% of the variance in overall comfort, and that the overall model demonstrated statistically significant explanatory power. Both TCV and ACV had positive regression coefficients, indicating that, after controlling for the other environmental comfort variable, improvements in thermal and acoustic comfort were positively associated with higher overall comfort. The model consists of two subjective comfort indicators with clear environmental meanings, resulting in a simple and interpretable structure. It can therefore serve as a fundamental model for subsequent analysis of the thermal–acoustic coupling effects and overall environmental comfort of elevated bridge underpass spaces. The PET–TSV–TCV and L_Aeq_–ASV–ACV relationships were subsequently summarized as a conceptual framework to aid interpretation. No mediation or structural equation modeling was performed; therefore, these pathways were not interpreted as statistically verified causal mechanisms.

### 4.5. Numerical Simulation Setup and Validation

**Figure 17:**
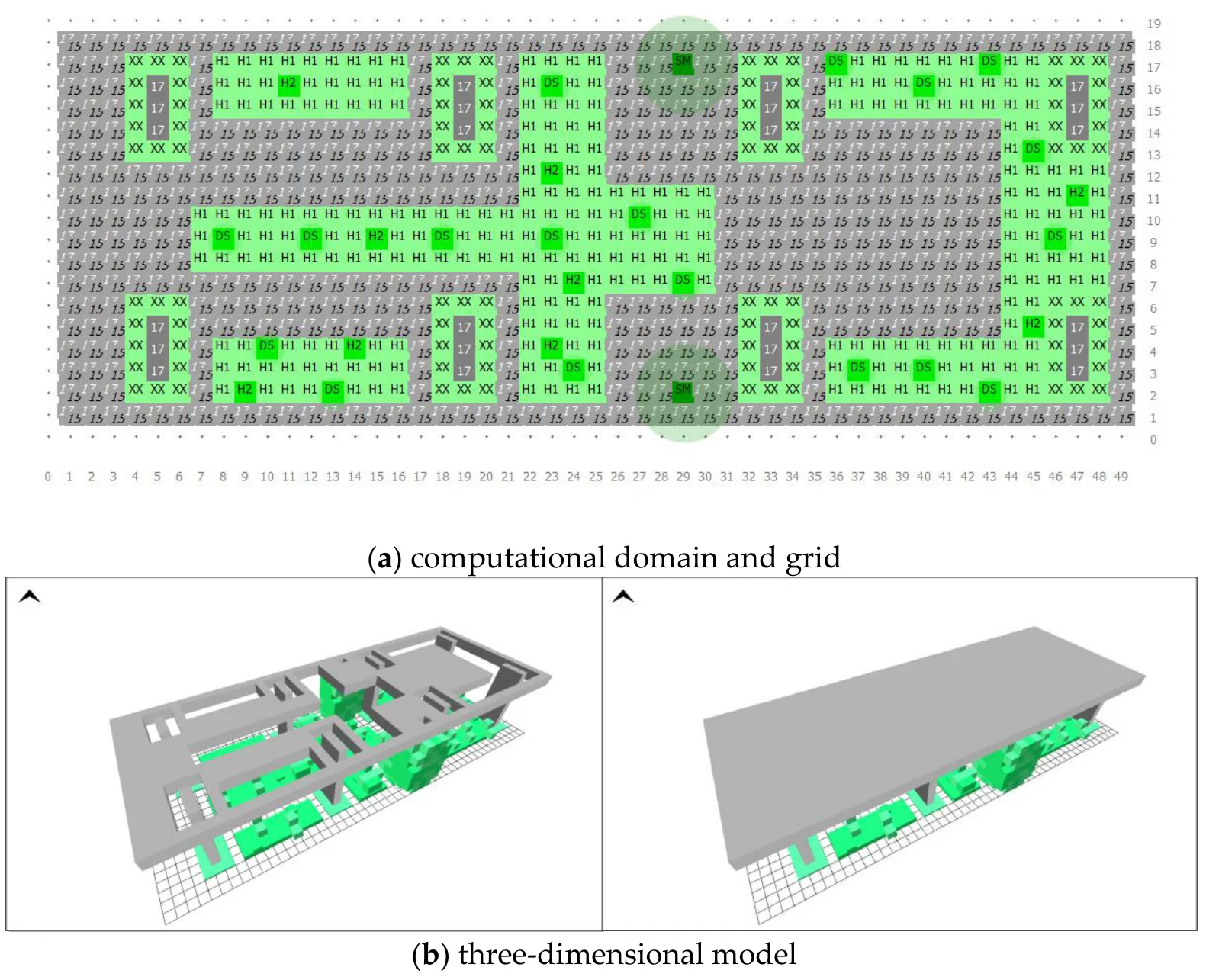
ENVI-met model configuration of the representative elevated bridge underpass space.

ENVI-met (Version 5.9.0) was used to simulate the thermal environment of the Minjiang Park section of Youxizhou Bridge. The model domain was discretized into 50 × 20 horizontal grids with a 3 m resolution (150 m × 60 m) and 15 vertical layers at 1 m intervals. The bridge geometry, major ground surfaces, and vegetation structure were represented according to field measurements. Simulations were conducted for 2 July 2025 from 08:00 to 18:00 using measured air temperature and relative humidity, a reference wind speed of 4.5 m/s at 10 m, and a prevailing southerly wind. Outputs were recorded hourly at 1.5 m above ground, with PET, air temperature, mean radiant temperature, and wind speed used as the main indicators. The current and optimized scenarios differed mainly in green coverage (28% vs. 40%), hard paving proportion (62% vs. 50%), tree canopy width (3.0 vs. 4.5 m), shrub-layer height (0.6 vs. 1.1 m), and green buffer width (1.5 vs. 4.5 m) (Figure 17).

**Figure 18:**
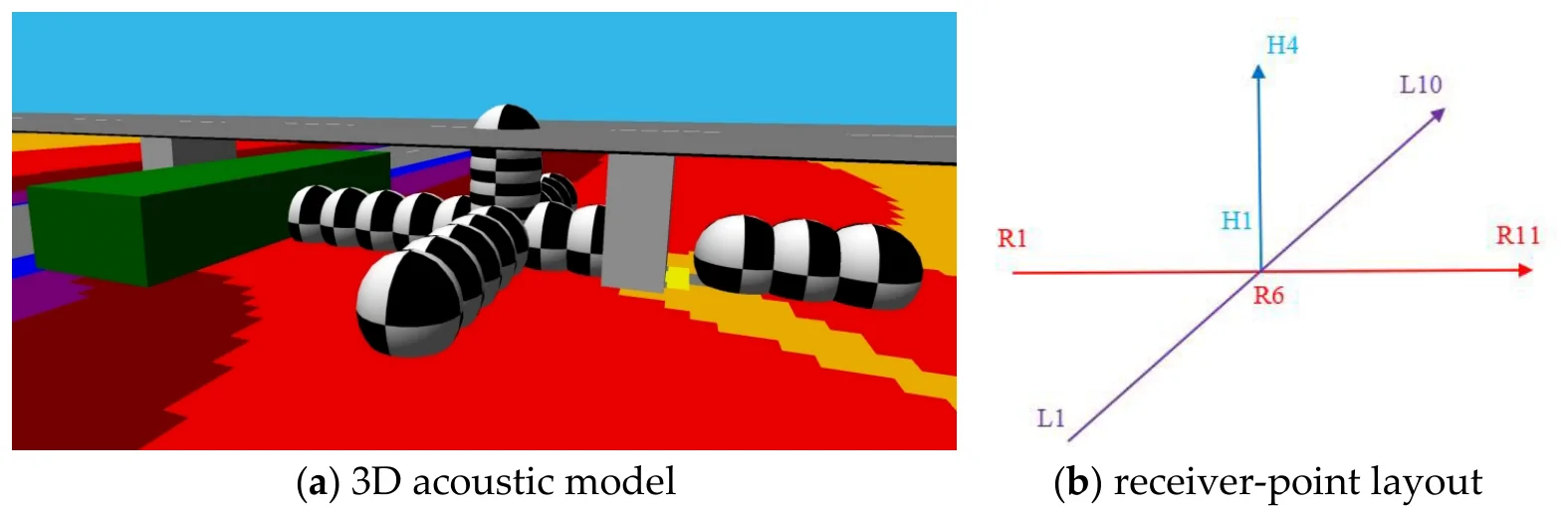
Cadna/A model configuration and receiver-point layout.

Cadna/A (Version 2026 MR1) was used to simulate daytime traffic noise propagation at the Wenping Road elevated bridge underpass space (Figure 18). The model covered a 45 m × 25 m × 6.5 m domain and included the bridge, adjacent road, ground surfaces, and roadside green buffer zones. The adjacent urban arterial was represented as a two-way, four-lane road with a width of 16 m, a speed of 40 km/h, and approximately 15,600 vehicles during 08:00–18:00. Green-buffer widths of 0, 5, and 8 m were simulated using a mixed tree-shrub configuration with a uniform vegetation height of 4 m. Twenty-five receiver points were distributed across near-road, central, and far-road locations. A 2 m calculation grid and a receiver height of 1.5 m were adopted, and L_Aeq_ was used as the primary acoustic indicator. The calculation followed ISO 9613-2 [65] and the RLS90 road-traffic noise model. Model inputs were derived from field surveys, traffic volume records, and site drawings to reproduce the existing spatial and traffic conditions. Because corresponding point-level measured and simulated L_Aeq_ data were not available, the Cadna/A model was used for comparative scenario analysis rather than quantitative validation or absolute noise prediction.

## 5. Conclusions

This study investigated the coupled thermal–acoustic comfort of 12 elevated bridge underpass spaces in Fuzhou and Xiamen and identified the main structural and landscape factors associated with environmental comfort. The main conclusions are as follows:

(1) Thermal–acoustic conditions were strongly associated with overall comfort. Thermal and acoustic comfort jointly explained 71.3% of the variance in OCV (*R*^2^ = 0.713), with thermal comfort contributing more strongly. The observed relationships were summarized by the conceptual “PET-TSV-TCV” and “L_Aeq_-ASV-ACV” pathways.
(2) Bridge orientation and landscape configuration were associated with differences in overall comfort. East–west-oriented elevated bridge underpass spaces generally provided better comfort due to more stable shading. Across orientations, green coverage rate, green buffer width, and distance from roads were positively associated with OCV, whereas hard paving proportion showed a negative association with OCV. Tree canopy width and multi-layer vegetation showed stronger effects in north–south and northeast–southwest-oriented spaces.
(3) Within the sampled Fuzhou and Xiamen sites, better comfort was associated with 40–55% green coverage, 30–50% hard paving, a road distance greater than 60 m, and an 8–15 m green buffer. In north–south and northeast–southwest-oriented spaces, a tree crown width of approximately 2.5–3.5 m was also associated with better comfort. These values should be regarded as context-specific design references for hot–humid subtropical under-bridge spaces rather than universal optimal thresholds. Numerical simulations further provided scenario-based evidence for the potential thermal and acoustic benefits of landscape optimization.

## Figures and Tables

**Table 1:** Correlations between environmental parameters and subjective environmental perceptions.

Perception	T_a_	RH	V_a_	MRT	PET	L_Aeq_
TSV	0.453 **	0.296 **	−0.115 *	0.423 **	0.621 **	0.201 **
TCV	−0.268 **	−0.251 **	0.103	−0.276 **	−0.637 **	−0.213 **
TPV	−0.342 **	−0.208 **	0.256 **	−0.458 **	−0.586 **	−0.148
ASV	0.031 *	0.035	−0.037	0.072 *	0.101 *	−0.583 **
ACV	−0.033 *	−0.028	0.028	−0.175 *	−0.187 *	−0.601 **
APV	−0.112 *	−0.108	0.056	−0.159 *	−0.126 *	−0.454 **
OCV	−0.316 **	−0.135 *	0.108 *	−0.355 **	−0.437 **	−0.401 **

Note: ** represents *p* (two-tailed) < 0.01, * represents *p* (two-tailed) < 0.05.

**Table 2:** Correlation analysis of thermal comfort, acoustic comfort and overall comfort.

Variable	TCV	ACV	OCV
TCV	1	0.24 **	0.76 **
ACV	0.24 **	1	0.54 **
OCV	0.76 **	0.54 **	1

Note: TCV is thermal comfort vote, ACV is acoustic comfort vote, OCV is overall comfort vote; it was calculated using Spearman rank correlation analysis, indicating a significant correlation at the 0.01 level (*p* < 0.01). ** represents *p* (two-tailed) < 0.01.

**Table 3:** Subgroup analysis of the associations between TCV, ACV, and OCV.

Factor	Subgroup	*n*	TCV-OCV (ρ)	*p*	ACV-OCV
Gender	Male	780	0.79	<0.001	0.61
	Female	800	0.78	<0.001	0.60
Age	≤50 years	720	0.77	<0.001	0.59
	>50 years	860	0.80	<0.001	0.62
Outdoor stay duration	≤30 min	300	0.76	<0.001	0.58
	>30 min	1280	0.79	<0.001	0.61

**Table 4:** Information table on morphological characteristics of underground space of bridges.

Full Name	Abbreviation	Move Towards	Clear Height (m)	Bridge Deck Width (m)	Width-to-Height Ratio (W/H)	Pier Spacing (m)	Adjacent Road Condition
The underpass space of the Jiu Yuanzhou Expressway Construction Phase	GY	East–west direction	10.0	40	4.00	30	Non-adjacent type
The underpass space of Qing Ting Lake Elevated Bridge	QT	North–south direction	10.0	25	2.50	25	Adjacent type
The underpass space of You Xi Zhou Bridge at Minjiang Park section	YX	North–south direction	15.0	36	2.40	30	Non-adjacent type
The underpass space of Gushan Bridge at Huai Hai Park section	GS	Northeast–southwest direction	25.0	40	1.60	40	Non-adjacent type
The underpass space of Ku Pu Bridge	KP	Northeast–southwest direction	20.0	38	1.90	50	Non-adjacent type
The underpass space of San Xian Zhou Bridge	SX	North–south direction	8.5	32	4.00	55	Non-adjacent type
The underpass space of Cheng Gao Da Road section of Gao Lin Road	LL	North–south direction	6.5	30	4.60	25	Adjacent type
The underpass space of Wen Ping Road Elevated Bridge	WP	Northeast–southwest direction	5.5	18	3.30	25	Adjacent type
The underpass space of Yan Wu Bridge at Cheng Gao Da Road section	YW	East–west direction	5.0	20	4.00	20	Non-adjacent type
The underpass space of Wuyuanwan Bridge	WY	East–west direction	8.5	32	3.80	40	Non-adjacent type
The underpass space of Haicang Bridge at Daping Mountain Rural Park Section 1	HC1	East–west direction	20.0	35	1.75	40	Non-adjacent type
The underpass space of Haicang Bridge at Daping Mountain Rural Park Section 2	HC2	East–west direction	10.0	35	3.50	40	Non-adjacent type

**Table 5:** Statistical table of environment and comfort for the entire sample.

Abbreviation	OCV	PET (°C)	L_Aeq_ [dB(A)]	T_a_ (°C)	T_g_ (°C)	V_a_ (m/s)	RH (%)
GY	−0.12	34.6	68.9	32.3	34.0	1.3	64.5
QT	−0.46	36.6	69.3	33.9	34.8	0.6	69.5
YX	−0.28	35.4	68.0	33.0	35.2	2.8	67.0
GS	−0.24	35.2	69.9	32.4	34.7	0.5	65.5
KP	−0.34	36.4	67.9	34.1	34.5	0.5	65.0
SX	−0.38	36.9	69.6	33.5	38.0	0.7	65.5
LL	−0.58	36.1	72.2	33.2	34.8	0.2	65.5
WP	−0.42	35.9	69.7	32.4	35.3	0.3	72.0
YW	−0.10	34.1	68.3	31.8	35.0	0.1	70.0
WY	−0.14	34.4	70.8	31.8	34.0	1.2	67.0
HC1	−0.05	33.9	67.2	33.0	33.5	0.3	67.5
HC2	0.12	32.7	64.9	31.7	33.2	0.3	69.0

**Table 6:** Spearman correlation matrix among candidate micro-landscape variables.

Variables	GCR	AHSL	AHTL	SPC	CSTS	GER	PHSP	PSHC	PRC	SR	DFWB	BER	DFR
GCR	1.000	-	-	-	-	-	-	-	-	-	-	-	-
AHSL	0.463	1.000	-	-	-	-	-	-	-	-	-	-	-
AHTL	0.356	0.574	1.000	-	-	-	-	-	-	-	-	-	-
SPC	0.512	0.612	0.638	1.000	-	-	-	-	-	-	-	-	-
CSTS	0.681	0.501	0.472	0.547	1.000	-	-	-	-	-	-	-	-
GER	0.528	0.318	0.284	0.426	0.455	1.000	-	-	-	-	-	-	-
PHSP	−0.693	−0.352	−0.286	−0.415	−0.594	−0.487	1.000	-	-	-	-	-	-
PSHC	−0.116	−0.041	−0.023	−0.036	−0.082	−0.063	0.124	1.000	-	-	-	-	-
PRC	0.084	0.062	0.055	0.071	0.096	0.052	−0.092	0.356	1.000	-	-	-	-
SR	0.173	0.214	0.185	0.201	0.238	0.176	−0.215	0.284	0.418	1.000	-	-	-
DFWB	0.214	0.137	0.105	0.128	0.174	0.186	−0.176	−0.073	−0.041	−0.086	1.000	-	-
BER	−0.102	−0.083	−0.071	−0.094	−0.115	−0.138	0.162	0.046	0.032	0.112	−0.284	1.000	-
DFR	0.186	0.096	0.082	0.114	0.174	0.158	−0.214	−0.061	−0.054	−0.102	0.347	−0.356	1.000
GBW	0.528	0.291	0.246	0.337	0.416	0.492	−0.487	−0.102	0.073	0.154	0.612	−0.218	0.612

**Table 7:** Spearman correlation analysis of micro-landscape elements and OCV along the north–south/east–northwest direction group.

Microscopic Landscape Elements	Coefficient of Association (ρ)
Green coverage rate	0.479 **
Average height of shrub layer	0.450 **
Average height of tree layer	0.269
Community structure	0.399 **
Tree canopy spread	0.486 **
Greening enclosure degree	0.398 **
Proportion of hard paving	−0.423 **
Pavement specific heat capacity	0.064
Pavement reflection coefficient	−0.018
Pavement roughness	−0.240
Distance from water body	0.116
Building enclosure degree	−0.248
Distance from road	0.336 *
Greening buffer width	0.516 **

Note: * *p* < 0.05, ** *p* < 0.01. Positive values indicate that the factor increases when OCV increases overall, and negative values indicate that the factor increases when OCV decreases overall.

**Table 8:** Spearman correlation analysis of micro-landscape elements and OCV in the east–west orientation group.

Microscopic Landscape Elements	Coefficient of Association (ρ)
Green coverage rate	0.545 **
Average height of shrub layer	0.312
Average height of tree layer	0.149
Community structure	0.205
Tree canopy spread	0.027
Greening enclosure degree	0.086
Proportion of hard paving	−0.578 **
Pavement specific heat capacity	0.303
Pavement reflection coefficient	0.083
Pavement roughness	−0.325
Distance from water body	0.381 *
Building enclosure degree	−0.282
Distance from road	0.400 *
Greening buffer width	0.378 *

Note: * *p* < 0.05, ** *p* < 0.01. The positive values indicate that the overall OCV shows an upward trend when the factor increases, while the negative values indicate that the overall OCV shows a downward trend when the factor increases.

**Table 9:** Results of the control regression analysis of OCV by common key continuous factors (Model B).

Independent Variable	Unstandardized Coefficient B	Standard Error	Standardized Coefficient Beta	t	*p*	VIF
Constant	0.178	0.143	-	1.244	0.218	-
Proportion of hard surface pavement	−0.016	0.002	−0.628	−8.377	<0.001	2.433
Distance from the road	0.007	0.001	0.515	7.125	<0.001	2.336
Green buffer width	0.046	0.010	0.336	4.722	<0.001	1.487

Note: The dependent variable is OCV. Model B is the surface control model, which includes the proportion of hard surface pavement, the distance from the road, and the width of the green buffer. *R*^2^ = 0.684, adjusted *R*^2^ = 0.670, *F* = 49.123, *p* < 0.001. VIF is used for multicollinearity diagnosis.

**Table 10:** Quantitative validation of ENVI-met thermal simulations.

Monitoring Point	T_a_-RMSE (°C)	T_a_-MAPE (%)	RH-RMSE (%)	RH-MAPE (%)
Point 1	0.46	1.31	1.20	1.84
Point 2	0.33	0.87	0.89	1.32
Point 3	0.22	0.55	0.63	0.94
Mean	0.33	0.91	0.91	1.37

**Table 11:** Two-way ANOVA results for OCV with PET and L_Aeq_ as factors.

Overall Perception Evaluation	PET	L_Aeq_	Interaction
OCV	<0.001 ***	<0.001 ***	0.013 *

Note: * *p* < 0.05, *** *p* < 0.001.

**Table 12:** Thermal environment monitoring instruments and technical parameters.

Instrument (Model)	Measurement Parameters	Measurement Range	Measurement Accuracy
HOBO S-THC-M002	Air temperature (T_a_)	−40–75 °C	±0.25 °C
	Relative humidity (RH)	0–100%	±2.5%
HOBO S-WCF-M003	Wind speed (V_a_)	0–76 m/s	±1.1 m/s
HOBO S-LIB-M003	Solar radiation intensity (G)	0–1280 W/m^2^	±10 W/m^2^
JT2011	Blackbody temperature (T_g_)	0–120 °C	±0.5 °C

**Table 13:** Measurement dates and weather conditions for the field surveys.

Sample	Date	Maximum Air Temperature (°C)	Weather	Wind Speed
GY	1 July 2025	35.8	Clear	N, Force 1
YX	2 July 2025	36.8	Cloudy	N, Force 1
GS	3 July 2025	35.6	Mostly cloudy	N, Force 1
QT	8 August 2025	37.6	Clear	SE, Force 1
KP	9 August 2025	37.8	Cloudy	SE, Force 2
SX	10 August 2025	37.7	Clear	SE, Force 2
LL	21 August 2025	36.9	Cloudy	SE, Force 1
WP	22 August 2025	36.8	Clear	SE, Force 1
YW	24 August 2025	36.8	Cloudy	SW, Force 1
WY	25 August 2025	36.9	Clear	SE, Force 1
HC1	26 August 2025	35.9	Clear	E, Force 2
HC2	27 August 2025	35.7	Cloudy	NE, Force 1

**Table 14:** Contents of the subjective perception evaluation in the questionnaire and scale settings.

Numerical Score	−4	−3	−2	−1	0	1	2	3	4
Temperature	Extremely cold	Cold	Cool	Slightly cool	Moderate	Slightly warm	Warm	Hot	Extremely hot
Numerical score	−3		−2	−1	0	1	2	3	
Sound	Very loud		Loud	A little louder	Moderate	A little quieter	Silence	Very quiet	
Sound comfort	Very uncomfortable		Uncomfortable	A little uncomfortable	Moderate	A little more comfortable	Comfortable	Very comfortable	
Thermal comfort	Very uncomfortable		Uncomfortable	A little uncomfortable	Moderate	A little more comfortable	Comfortable	Very comfortable	
Overall comfort	Very uncomfortable		Uncomfortable	A little uncomfortable	Moderate	A little more comfortable	Comfortable	Very comfortable	

**Table 15:** Fitting analysis of overall comfort.

Dependent Variable	Independent Variable	Linear Model	Quadratic Model	Cubic Model
OCV	TCV	0.578 **	0.596 **	0.603 **
	ACV	0.292 **	0.308 **	0.314 **

Note: ** represents *p* (two-tailed) < 0.01.

## Data Availability

The original contributions presented in the study are included in the article; further inquiries can be directed to the corresponding authors.

## Supplementary Materials

The following supporting information can be downloaded at: https://www.mdpi.com/article/10.3390/plants15192932/s1. Figure S1: Layout of space measurement points under Juyuan Bridge in Fuzhou; Figure S2: Arrangement of space measurement points under Qinting Lake Bridge in Fuzhou; Figure S3: Layout of space measurement points under Youxizhou Bridge in Fuzhou; Figure S4: Layout of space measurement points under Gushan Bridge in Fuzhou; Figure S5: Layout of space measurement points under Kuipu Bridge in Fuzhou; Figure S6: Layout of space measurement points under Sanxian Zhou Bridge in Fuzhou; Figure S7: layout of space measurement points under Cheng Gao Da Road section of Gao Lin Road in Xiamen; Figure S8: Layout of space measurement points under the bridge of Wenping Road in Xiamen; Figure S9: Layout of space measurement points under Yanwu Bridge in Xiamen; Figure S10: Layout of space measurement points under Wuyuanwan Bridge in Xiamen; Figure S11: Layout of space measurement points under the first section of Haicang Bridge in Xiamen; Figure S12: Layout of space measurement points under the second section of Haicang Bridge in Xiamen.

## Abbreviations

The following abbreviations are used in this manuscript:

PMV	Predicted Mean Vote
PET	Physiologically Equivalent Temperature
UTCI	Universal Thermal Climate Index
L_Aeq_	A-weighted equivalent continuous sound pressure level
T_a_	Air temperature
RH	Relative humidity
V_a_	Wind speed
G	Solar radiation intensity
T_g_	Globe temperature
MRT	Mean radiant temperature
TSV	Thermal Sensation Vote
ASV	Acoustic Sensation Vote
TCV	Thermal Comfort Vote
ACV	Acoustic Comfort Vote
TPV	Thermal Preference Vote
APV	Acoustic Preference Vote
OCV	Overall Comfort Vote
GCR	Green coverage ratio
AHSL	Average height of the shrub layer
AHTL	Average height of the tree layer
SPC	Structure of plant community
CSTS	The crown spread of a tree species
GER	Greening enclosure ratio
PHSP	Proportion of hard surface paving
PSHC	Pavement specific heat capacity
PRC	Pavement reflection coefficient
SR	Surface roughness
DFWB	Distance from water bodies
BER	Building enclosure ratio
DFR	Distance from the road
GBW	Greening buffer width
