# Supplementary material for: Microclimate Comfort of Elevated Bridge Underpass Spaces Based on Sound and Thermal Environments: A Case Study of Fuzhou and Xiamen, Fujian Province
Source: Plants (Basel). 2026 Sep 24;15(19):2932. doi: 10.3390/plants15192932 (PMC13667106; doi:10.3390/plants15192932)
Supplement: Supplementary file 1 [file plants-15-02932-s001.zip › plants-4556879-supplementary.pdf]

Supplementary Materials：Layout of Measuring Points in Underpass Space

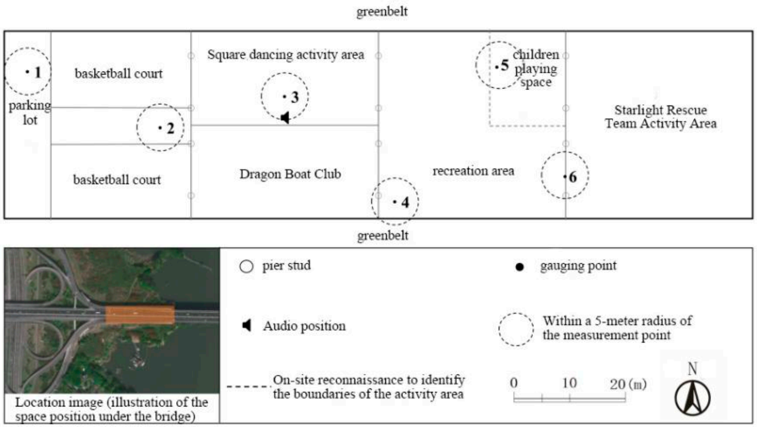

Figure S1 Layout of space measurement points under Juyuan Bridge in Fuzhou

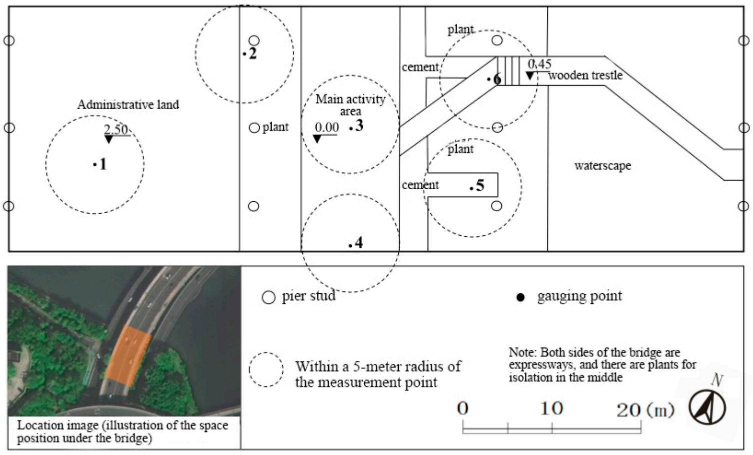

Figure S2 Arrangement of space measurement points under Qinting Lake Bridge in Fuzhou

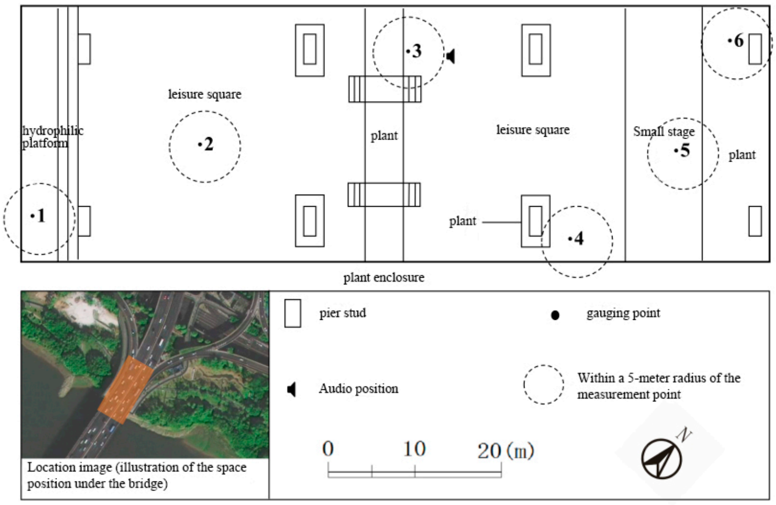

Figure S3 Layout of space measurement points under Youxizhou Bridge in Fuzhou

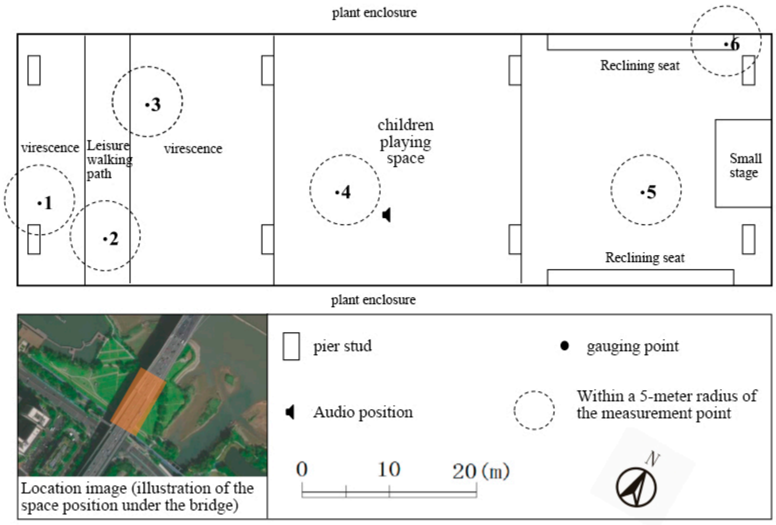

Figure S4 Layout of space measurement points under Gushan Bridge in Fuzhou

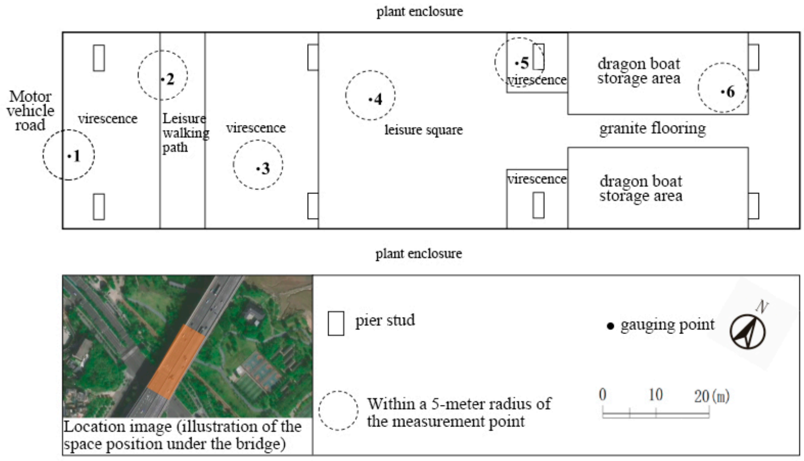

Figure S5 Layout of space measurement points under Kuipu Bridge in Fuzhou

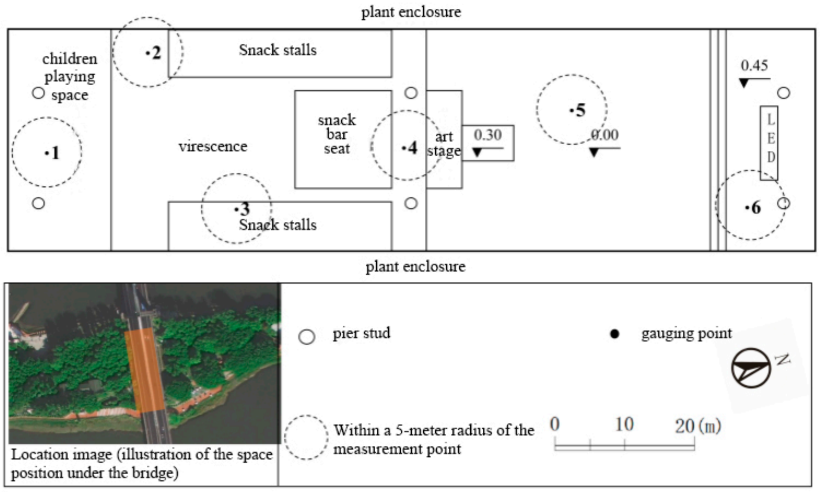

Figure S6 Layout of space measurement points under Sanxian Zhou Bridge in Fuzhou

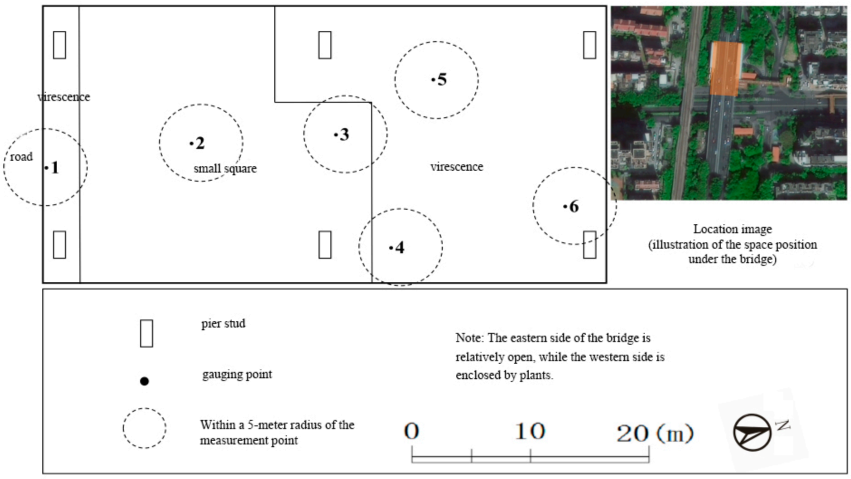

Figure S7 layout of space measurement points under Cheng Gao Da Road section of Gao Lin Road in Xiamen

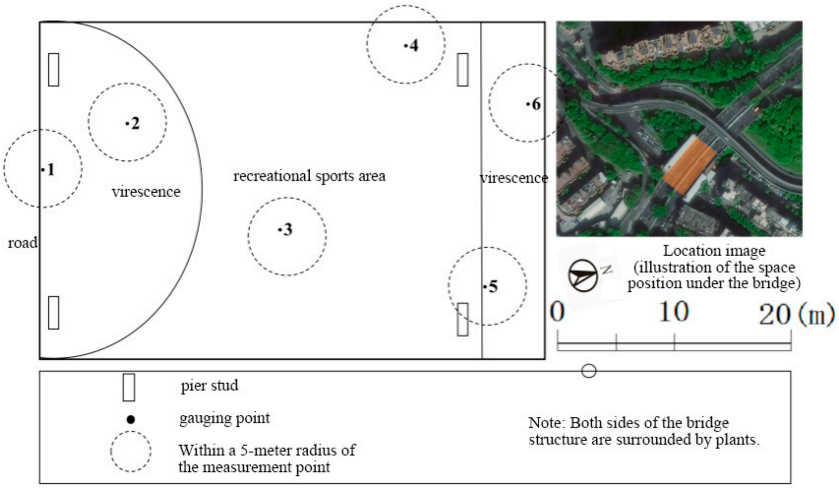

Figure S8 Layout of space measurement points under the bridge of Wenping Road in Xiamen

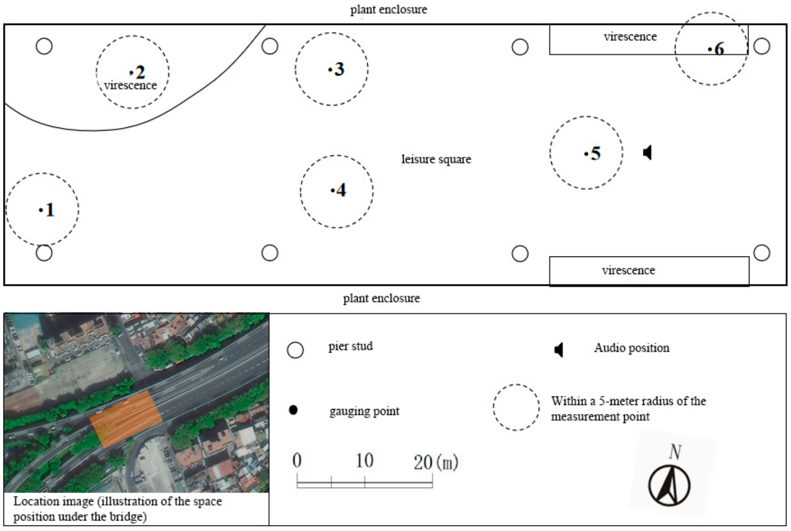

Figure S9 Layout of space measurement points under Yanwu Bridge in Xiamen

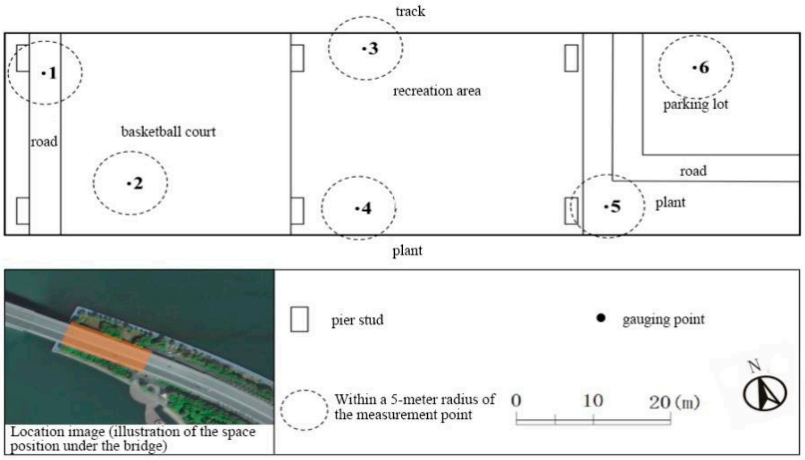

Figure S10 Layout of space measurement points under Wuyuanwan Bridge in Xiamen

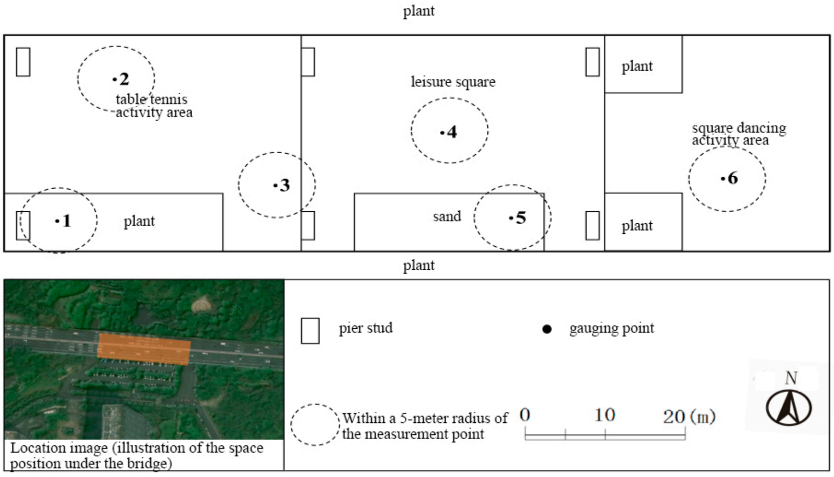

Figure S11 Layout of space measurement points under the first section of Haicang Bridge in Xiamen

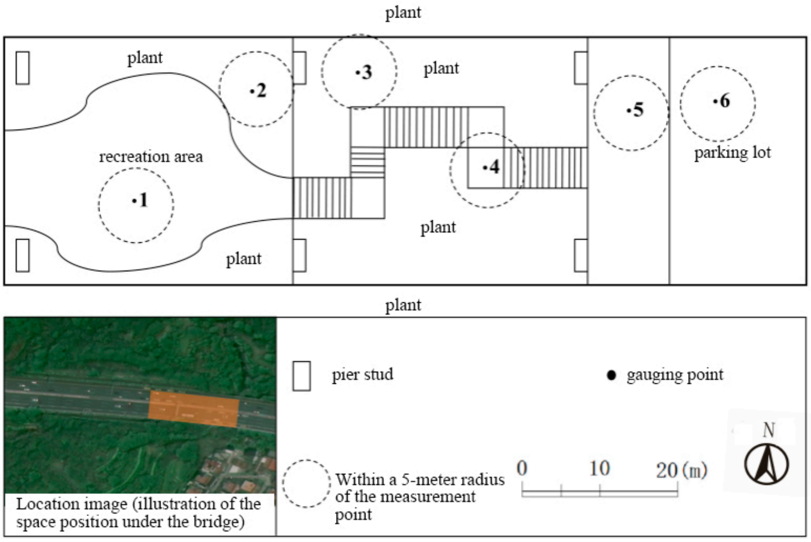

Figure S12 Layout of space measurement points under the second section of Haicang Bridge in Xiamen
